# Understanding how social determinants of health shape Long COVID outcomes: a rapid review of evidence

**DOI:** 10.1186/s13690-025-01787-x

**Published:** 2025-12-29

**Authors:** Tala Tamim El Jarkass, Shankavi Nandakumar, Becky Skidmore, Andrew D. Pinto, Banafshe Hosseini

**Affiliations:** 1 Upstream Lab, MAP Centre for Urban Health Solutions, Li Ka Shing Knowledge Institute, St. Michael’s Hospital, Unity Health Toronto, 30 Bond Street, Toronto, ON M5B 1W8 Canada; 2 Department of Human Biology, Faculty of Arts and Science, University of Toronto, Toronto, ON Canada; 3 Independent Information Specialist, Ottawa, ON Canada; 4 Department of Family and Community Medicine, Faculty of Medicine, University of Toronto, Toronto, ON Canada; 5 Division of Clinical Public Health, Dalla Lana School of Public Health, University of Toronto, Toronto, ON Canada

**Keywords:** Social determinants of health, COVID, Long COVID

## Abstract

**Background:**

Long COVID affects over 65 million people worldwide, yet the impact of social determinants of health (SDoH), such as socioeconomic status, race/ethnicity, education, occupation, and geography, remains poorly understood. To evaluate the association between SDoH and the risk and severity of Long COVID.

**Methods:**

A rapid review of observational studies was conducted using MEDLINE, Embase, and Web of Science (up to September 29, 2024). Studies reporting original data on SDoH and Long COVID outcomes were included. Data were extracted on study characteristics, population demographics, Long COVID definitions, and SDoH-related findings. Study quality was assessed using the Newcastle-Ottawa Scale.

**Results:**

Seventy-one studies (43 cohort, 28 cross-sectional) were included. Definitions of Long COVID varied. Commonly studied SDoH included age, sex, race/ethnicity, education, financial security, employment, and geography. Female sex and older age were consistently associated with increased risk and severity of Long COVID. Black and Hispanic individuals were more likely to experience Long COVID. Lower education and financial insecurity were also linked to greater prevalence and symptom burden. Frontline and essential workers were found to be at increased risk. Geographic disparities were evident but varied across rural and urban residence.

**Conclusions:**

SDoH play a key role in shaping Long COVID outcomes. Addressing these disparities requires targeted public health efforts and standardized case definitions.

**Supplementary Information:**

The online version contains supplementary material available at 10.1186/s13690-025-01787-x.

**Table:** 

Text box 1. Contributions to the literature
• Synthesizes current evidence on the association between social determinants of health and long COVID incidence, severity, and recovery.
• Identifies key social and structural factors impacting disparities in long COVID outcomes.
• Highlights gaps in existing research, including underrepresentation of certain populations and inconsistent measurement of SDoH.

## Background

Since its emergence in late 2019, SARS-CoV2 has posed significant challenges to the public health system, and highlighted health disparities. While the acute clinical manifestations of SARS-CoV2 range from asymptomatic infection to severe respiratory failure and multi-organ dysfunction, evidence shows that patients may experience persistent post-infectious symptoms (e.g., fatigue, brain fog, chest or throat pain, dyspnea) for over two months [1, 2]. Although prevalence rates vary, these symptoms affect approximately 50% of non-hospitalized patients and up to 87% of hospitalized patients. The Centers for Disease Control and Prevention (CDC) refers to SARS-CoV2 symptoms lasting over four weeks as “Post-Acute Sequelae of SARS-CoV2” or Long COVID [3]. As of 2023, it is estimated that at least 65 million individuals globally are suffering from Long COVID, with the number of cases rising daily [4, 5]. Despite seeking medical care, only about 12.5% who sought help felt they received adequate care [6]. This underscores the significant impact of Long COVID and highlights a substantial gap in healthcare provision for those suffering from Long COVID.

Epidemiological studies in the United States [7, 8], Canada [9–11], and the United Kingdom [12] have consistently shown that SARS-CoV2 disproportionately impacts communities with higher densities of low-income households, significant overcrowding, and a high prevalence of racialized individuals [7–13]. These findings underscore the vulnerability of these groups perhaps not only to initial infection, but to its prolonged aftermath. While existing literature has explored the impact of social determinants of health (SDoH) on SARS-CoV2 incidence and outcomes [9–11], evidence remains limited on how factors such as occupation, educational attainment, housing status, food security, and area of residence impact the prevalence of Long COVID [14]. Several studies have demonstrated that certain factors such as sex, age, and pre-existing health conditions may increase the risk of developing Long COVID [15–19], but the role of social determinants of health is less clear and often limited to specifics areas such as occupational differences and financial impact [20, 21]. As SARS-CoV2 transitions to its endemic phase, the virus will continue to spread, and Long COVID will persistently affect individuals, especially those in marginalized populations that often face barriers to healthcare and social support. Resultantly, it is critical to understand the nuances of Long COVID in this new context to inform equitable healthcare practices and guide effective public health responses [14]. 

To address this knowledge gap, this rapid review aims to evaluate the association between social determinants of health and both the risk and severity of Long COVID. By examining key social factors, this review seeks to provide insights into how these determinants influence Long COVID outcomes and identify potential areas for targeted interventions.

## Methods

### Search strategy and eligibility criteria

The protocol was published on Open Science (ID: S3MKY). A senior information specialist (BS) developed the search strategy in consultation with the review team. The MEDLINE search strategy was reviewed by another experienced information specialist prior to execution using the Peer Review of Electronic Search Strategies (PRESS) checklist [22]. Using the multifile option and deduplication tool in Ovid, we searched Ovid MEDLINE^®^ ALL and Embase Classic + Embase. The Web of Science (core databases, excluding conference databases) was also searched. The strategies used a combination of subject headings (e.g., “Post-Acute COVID-19 Syndrome”, “Social Determinants of Health”, “Educational Status”) and keywords (e.g., long COVID, food access, academic background). An observational study design filter was applied. Vocabulary and syntax were adjusted across databases. Results were limited to publications published December 2019 or later and, where possible, animal only records, opinion pieces, case reports and conference abstracts were removed. All searches were performed on September 29, 2024. Records were downloaded and deduplicated using EndNote version 9.3.3 (Clarivate Analytics) and uploaded to Covidence (Veritas Health Innovation Ltd.**)** for screening. Please see Additional file 1 for the full search strategy.

Eligibility criteria for study inclusion were: (1) studies investigating the relationship between SDoH and Long COVID outcomes (2) studies reporting outcomes related to the incidence or severity of Long COVID; (3) observational studies, cohort studies, case-control studies, and cross-sectional studies; and (4) studies reporting original data. Inclusion was restricted to peer-reviewed publications in English. Exclusion criteria were: (1) Review articles and editorials; (2) non-peer-reviewed literature; and (3) studies focusing solely on acute COVID-19 outcomes.

### Study selection

Covidence was used to manage and screen all relevant publications. The study selection process involved three stages, conducted separately by two reviewers. Any discrepancies were resolved by consulting a third reviewer (BH). First level screening examined the titles and abstracts of all identified studies. The second stage involved reviewing the full text of selected studies to assess their eligibility regarding the primary and secondary outcomes of interest. Primary outcomes included the association between SDoH and the incidence, severity, and symptom burden of Long COVID, while secondary outcomes covered the frequency of Long COVID symptoms, healthcare access, and socioeconomic impact.

### Data collection and analysis

Extracted data included details such as author(s), title, journal, year of publication, study design, study population (e.g., country, age groups, demographics), sample size, reported SDoH, Long COVID outcomes, limitations, and implications for future research.

### Quality assessment

The methodological quality of the studies was assessed using the Newcastle-Ottawa Scale (NOS) [23], a widely accepted tool for evaluating the quality of non-randomized studies. Two reviewers assessed each eligible study. NOS evaluates studies based on: (1) the selection of study participants; (2) the comparability of groups based on exposure or outcome; and (3) the ascertainment of long COVID outcomes and SDoH variables. A star rating system is implemented which ranges from zero to nine, where studies with higher ratings are considered to have greater methodological quality [23]. Studies with seven to nine stars are categorized as high quality, four to six stars as moderate quality, and fewer than four stars as low quality. Please see Additional file 2 for the PRISMA 2020 Checklist.

### Data synthesis

A narrative synthesis review was employed to summarize the findings of the reviewed studies, focusing on the relationship SDoH and Long COVID outcomes. It was structured according to key SDoH variables such as sex, age race/ethnicity, education, income level, employment status, and occupation. These variables were examined in relation to long-term COVID incidence and prevalence, severity, and duration of symptoms. No quantitative pooling or subgroup comparisons were made when summarizing the findings.

The results were categorized to highlight trends, patterns, and disparities among various demographic groupings. Study findings were summarized based on their reported results, to provide a greater understanding of the ways in which each SDoH impacts the long-term course of COVID-19.

## Results

**Fig. 1:**
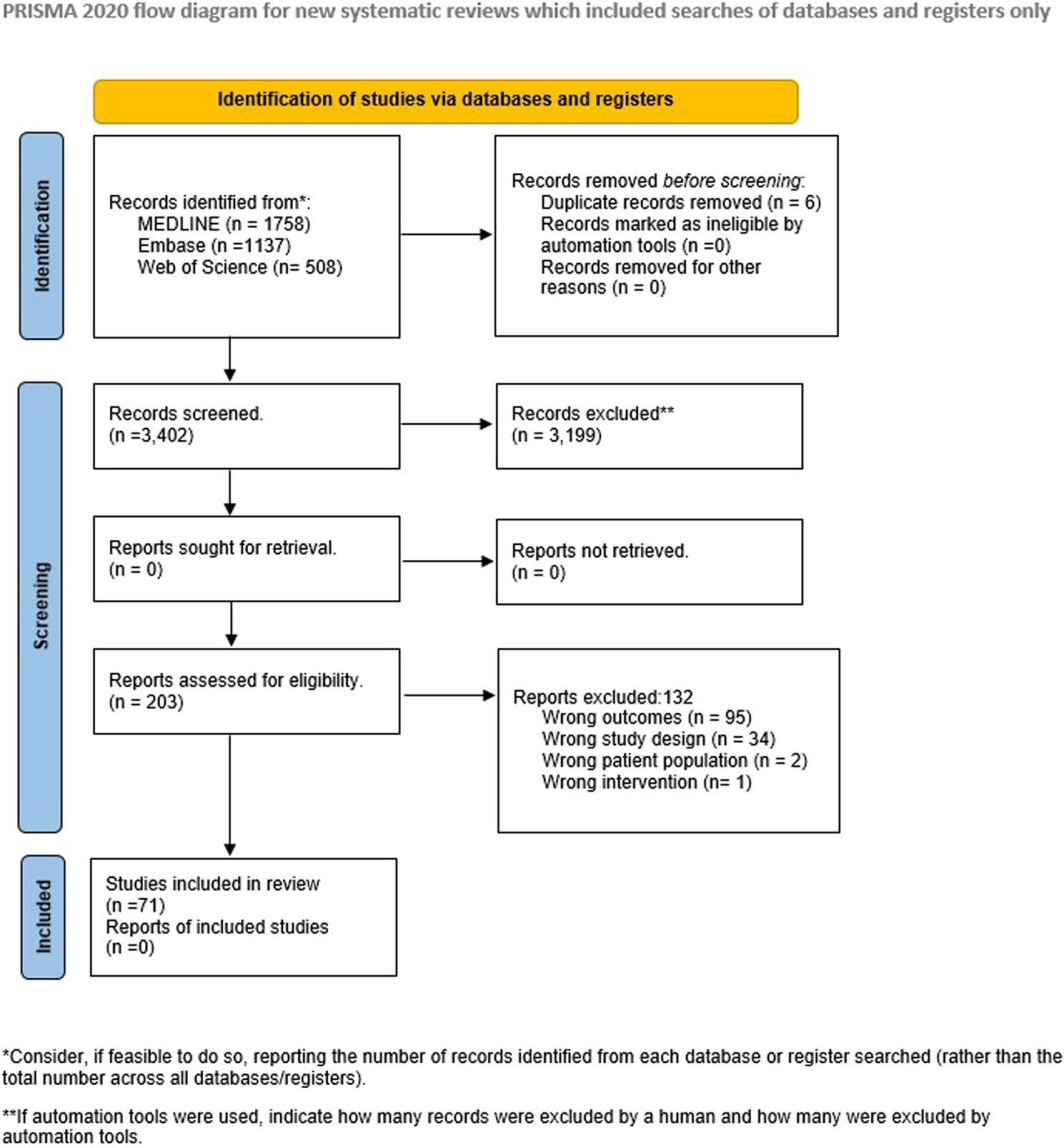
PRISMA flow diagram for the rapid review on social determinants of Long COVID

A total of 3,402 studies were screened at the title and abstract level, of which 203 studies progressed to full-text review. Finally, 71 studies were selected for data extraction after applying the inclusion and exclusion criteria (Fig. 1). Please see Additional file 3 for a summary of study characteristics and key findings. The final set included 43 cohort [24–66] and 28 cross-sectional [67–94] studies. Sample sizes in the studies varied from 48 participants in the smallest study [80] versus 19,462,260 participants in the largest [55]. The settings for these studies were geographically diverse and the age of study populations varied across studies. Please see Tables 1 and 2 for the geographic and age group distribution of the included studies.

**Table 1:** Geographic distribution of studies included in the rapid review on social determinants of Long COVID

Country	Number of Studies	References
United States	*25*	[26, 31, 34, 37, 38, 44, 45, 48–50, 57, 59, 60, 65, 71–73, 75, 76, 80, 83, 85–87, 92]
Brazil	6	[25, 40, 43, 58, 63, 79]
United Kingdom	5	[33, 36, 42, 55, 93]
Israel	4	[68, 78, 84, 91]
Italy	4	[30, 46, 64]
Japan	3	[35, 41, 77]
China	2	[69, 70]
France	2	[47, 89]
Multiple countries (Europe)	2	[32, 91]
Iran	2	[28, 81]
Spain	1	[67]
Mexico	1	[74]
South Africa	1	[29]
Denmark	1	[51]
Tunisia	1	[90]
Vietnam	1	[82]
Colombia	2	[52, 62]
Netherlands	1	[62]
Poland	1	[61]
Germany	1	[56]
Norway	1	[54]
Bangladesh	1	[53]
Suriname	1	[24]
Ghana	1	[88]
Malaysia	1	[94]
United Arab Emirates	1	[66]
Russia	1	[27]

Numbers indicate how many studies originated from each country, with corresponding reference numbers shown in the third column

**Table 2:** Age distribution of study populations included in the rapid review on social determinants of Long COVID

Age Group	Number of Studies	References
Adult populations (individuals aged 18 years and older)	60	-
Pediatric populations (individuals aged five to 17 years)	4	[68, 78, 82, 93]
Individuals aged 14 years and older	5	[42, 47, 50, 67, 81]
All ages	2	[31, 49]

The definition of Long COVID varied across studies. Table 3 outlines the different definitions of Long COVID found in the included studies.

**Table 3:** Definitions of Long COVID used in studies included in the rapid review on social determinants of health and Long COVID

Definitions of Long COVID	Number of Studies	References
Symptoms persisting longer than four weeks after initial SARS-CoV-2 infection	24	[26, 30, 31, 37, 39, 42, 48, 50, 51, 57, 59, 60, 65, 68, 71, 73, 75, 77, 79, 81, 84, 86–88]
Symptoms persisting for at least three months after infection	31	[24, 25, 27–29, 32, 33, 35, 36, 41, 46, 53, 54, 56, 58, 61–64, 67, 69, 72, 74, 78, 85, 90–94]
Symptoms persisting for at least two months after infection	3	[49, 76, 82]
Symptoms persisting for a minimum of six months after infection	2	[43, 47]
ICD-10 clinical coding used for classification	4	[34, 38, 44, 45]
SNOMED clinical coding for classification (standardized, machine-readable clinical terms used in electronic health records to document diagnoses, referrals, and assessments related to Long COVID)	1	[55]
Symptoms persisting longer than 28 days after infection	1	[66]
Multiple definitions applied (combinations of WHO, NICE, US-NCHS, and UK-ONS criteria)	1	[89]

*ICD-10* International classification of diseases, 10th revision, *SNOMED CT* Systematized nomenclature of medicine – clinical terms, *WHO* World health organization, *NICE* National institute for health and care excellence, *US-NCHS* United States national center for health statistics, *UK-ONS* United Kingdom office for national statistics

### Age and sex

All 71 studies (100%) examined the association between age and sex in relation to Long COVID. Age-related findings revealed heterogeneous results. 31/71 (44%) found a significant association between age and Long COVID risk with the majority concluding that older age was linked to an increased likelihood of developing Long COVID [30, 34, 35, 37, 38, 41, 43, 44, 49, 50, 54, 55, 71, 72, 74, 76, 90, 91]. However, 6/71 (8%) of these studies reported that adults under 65 years of age had a lower prevalence of Long COVID compared to younger adults [32, 36, 45, 51, 73, 85]. A few studies found that middle-aged individuals (typically between 35 and 64 years old) had a higher risk of developing Long COVID [35, 55, 72, 85]. Three out of 71 (4%) studies identified an inverse association between age and Long COVID symptoms [32, 46, 77]. One out of the three studies found that older age was linked to a lower number of symptoms [32], while the other two found that younger individuals were more likely to experience certain specific symptoms including fatigue, chest pain, palpitations, dysosmia, and dysgeusia [46, 77]. In contrast, 40/71 (56%) studies found no significant association between age and Long COVID.

Additionally, 52/71 (73%) studies found a significant association between sex and Long COVID, with most concluding that females were more likely to develop Long COVID compared to males [25, 27, 28, 30–36, 38, 42, 43, 47, 49, 51, 54–57, 60–62, 64, 66, 67, 72–74, 76, 85, 89, 90, 92, 94, 95], whereas 19/71 (27%) found no association. Multiple studies found that females were more likely to experience greater number, severity, and duration of Long COVID symptoms [24, 29, 40, 46, 52, 53, 58, 63, 69, 75–77, 81]. However, one study found that women were less likely to be diagnosed with Long COVID than men [88]. Findings from the 4/71 (6%) pediatric studies were generally consistent with those in adults. Most studies (75%) reported that older children had a higher risk of developing Long COVID compared to younger children [68, 78, 93]. However, one quarter (25%) of the studies found that children aged six to nine years had a higher risk of Long COVID compared to adolescents aged 10 to 16 years [82]. Similarly, one quarter (25%) of the studies found that girls were more likely to experience persistent symptoms [82], while another study found that males were less likely to have them [93]. 

### Race/ethnicity and education

Forty-two studies (59%) examined the association of race/ethnicity in relation to Long COVID. Among these, 25 (60%) found a significant association whereas, 17 (40%) found none. Fourteen (33%) studies reported that certain ethnic minority groups had a higher risk of developing Long COVID [33, 34, 36, 37, 40, 48, 50, 51, 62, 65, 66, 70, 83, 87]. Most of these studies identified a higher risk among Hispanic [34, 37, 48, 83] and Black [36, 37, 83] individuals. One study found that Black and Hispanic individuals were more likely to receive documented Long COVID care [45], while another found that they had lower awareness of Long COVID [86]. Two studies also reported that Black [65] and Hispanic [65, 87] individuals were more likely to experience certain, persistent Long COVID symptoms and related conditions over time. However, one study reported that Black participants had lower odds of reporting several common Long COVID symptoms, including loss of smell, wheezing, and other symptoms [48]. In contrast, 6 studies (14%) found a reduced likelihood of Long COVID in Black [34, 72, 73, 85], Hispanic [57, 73], and Asian [44, 72, 73] individuals.

Twenty-seven (38%) studies examined the relationship between education and Long COVID. Of these, fourteen (52%) found a significant association [32, 37, 47, 56, 63, 71, 72, 74, 76, 81, 86, 88, 91], whereas 13 (48%) found no association. The majority found that lower education levels were associated with a higher risk of Long COVID [47, 72, 88, 91], while higher education levels were associated with a reduced risk [32, 37, 76, 88]. However, two studies observed that people with higher levels of education were more likely to report symptoms of Long COVID [56, 81]. In contrast, a study of U.S. veterans found that higher education was protective, with those holding bachelor’s, master’s, or college degrees experiencing lower odds of moderate or severe symptoms [71]. 

### Financial security and employment status

Thirty-three (46%) studies examine the association between financial security and Long COVID. 15 (45%) found a significant association [26, 31, 33, 36, 42, 58, 59, 70, 73, 74, 80, 84–86, 93] whereas, the others (55%) found none. Most found that lower financial security, including lower income, socioeconomic status, and residing in more deprived areas, was associated with an increased risk and prevalence of Long COVID [36, 42, 43, 59, 70, 73, 74, 86]. A few studies also reported that individuals with lower socioeconomic status were more likely to experience Long COVID symptoms [26, 58, 84]. However, one study found a lower likelihood of Long COVID among individuals in the most deprived areas [33]. Another study highlighted that a household income above $100,000 per year was associated with reduced risk [85]. Moreover, one (3%) pediatric study reported that children in the most deprived areas were less likely to experience persistent symptoms [93]. 

Ten (14%) studies examine the relationship between employment status and Long COVID. Four (40%) studies found a significant association [31, 32, 43, 89] while the others (60%) found no association [41, 72, 75, 81, 86, 88]. Most studies found that unemployment was associated with a higher risk of Long COVID, with unemployed individuals experiencing greater incidence and prevalence of symptoms [31, 43, 89]. However, one study found a contradictory result, indicating that employment was associated with a greater risk [32]. 

### Geographical area and occupation

Nineteen (27%) studies examine the association of geographical area and Long COVID. Ten out of 19 (53%) studies found a significant association whereas [34, 37, 38, 45, 50, 53, 74, 76, 80, 83], 9/19 (47%) did not [37, 42, 67, 70–72, 75, 81, 82]. Two studies found that rural residence was associated an increased prevalence of Long COVID [53, 74]. Documentation of Long COVID (U09.9) was less common among individuals living in urban areas [34]. Another study found that urban veterans had a slightly higher risk of developing Long COVID compared to rural veterans [37]. One study reported increased odds of Long COVID among metropolitan workers compared to those in rural locations [50]. Urban residents were also more likely to seek Long COVID care [45]. However, one study found that urban residence was protective, as individuals living there had reduced odds [76]. Individuals in southern U.S. had higher odds of Long COVID symptoms [83]. Another study found that individuals experiencing Long COVID symptoms were less likely to reside in metropolitan areas [38]. 

Four (6%) studies examined the association between occupation and Long COVID, and all four studies (100%) found a significant association [42, 50, 53, 69]. Occupations significantly associated with Long COVID severity included frontline workers such as healthcare professionals and police officers [53]. Higher Long COVID risk was also observed in healthcare patient-facing roles [42]. Workers in manufacturing and public administration sectors were also at greater risk of developing Long COVID compared to individuals working in healthcare [50]. Employment in transportation, logistics, or disciplinary workforces was also associated with severe Long COVID [69]. 

### Quality assessments

The risk of bias assessment showed that most of the included studies were of high methodological quality. Tables 4 and 5 describe Quality Assessment Summary and Table 6 presents the detailed Risk of Bias Assessment. Of the 71 studies assessed using the Newcastle-Ottawa Scale, 56% (*n* = 40) were classified as high quality, 42% (*n* = 30) as moderate quality, and 1% (*n* = 1) as low quality. High quality studies were predominantly cohort designs.

**Table 4:** Quality assessment of studies included in the rapid review on social determinants of health and Long COVID

Study ID	Study design	Representativeness of exposed cohort	Selection of non-exposed cohort	Ascertainment of exposure	Outcome not present at start of study	Comparability	Assessment of outcomes	Length of follow-up	Adequacy of follow-up	Total
Adler et al., 2023	Cross-sectional	*	*	*		**				*****
Atchison et al., 2023	Cross-sectional	*	*			**				*****
Azambuja et al., 2024	Cohort	*	*	*	*	**		*	*	********
Bonner et al., 2024	Cross-sectional	*	*			**				****
Bovil et al., 2022	Cross-sectional	*	*			**				****
Chelly et al., 2023	Cross-sectional	*	*			**				****
Chilunga et al., 2023	Cohort	*	*	*	*	**	*	*	*	*********
Chudzik et al., 2022	Cohort	*	*	*		**		*	*	*******
Coste et al., 2024	Cross-sectional	*	*			*				****
Crankson et al., 2022	Cross-sectional	*	*	*		**				*****
Daniel et al., 2023	Cohort	*	*	*	*	**		*	*	*******
Durstenfeld et al., 2023	Cohort	*	*			**		*		*****
Eligulashvili et al., 2024	Cohort	*	*	*		*		*		*****
Feldstein et al., 2023	Cross-sectional	*	*			**				****
Ferreira et al., 2022	Cohort	*	*	*	*	**	*	*	*	*********
Feter et al., 2023	Cross-sectional	*	*			**				****
Evering et al., 2023	Cohort	*	*	*	*	**		*		*******
Fisher et al., 2024	Cross-sectional	*	*			**				****
Forster et al., 2022	Cohort	*	*	*	*	**		*		*******
Hejazian et al., 2024	Cross-sectional	*	*			**	*			*****
Heller et al., 2022	Cross-sectional	*	*			**				****
Henderson et al., 2024	Cohort	*	*	*	*	**	*	*	*	*********
Hetlevik et al., 2023	Cohort	*	*	*	*	**	*	*	*	*********
Hossain et al., 2021	Cohort	*	*	*	*	**		*	*	********
Krishnadath et al., 2023	Cohort	*	*	*	*	**		*		*******
Linh et al., 2024	Cross-sectional	*	*	*		**				*****
Mahmoodi et al., 2023	Cross-sectional	*	*			**				****
Martinez-Ayala et al., 2023	Cohort	*	*	*	*	**		*	*	*******
Martin et al., 2024	Cross-sectional		*	*		**				****
Paranhos et al., 2022	Cross-sectional	*	*	*		**	*			******
Merzon et al., 2022	Cross-sectional	*	*			**	*			******
Miyazato et al., 2022	Cross-sectional		*	*		**				***
Mkoma et al., 2024	Cohort	*	*	*	*	**	*	*	*	*********
Modji et al., 2024	Cohort	*	*	*	*	**	*	*		********
Mukherjee et al., 2022	Cohort	*	*	*	*	**	*	*		********
Laughlin et al., 2023	Cohort	*	*	*	*	**		*	*	********
Pastorello et al., 2025	Cohort	*	*	*	*	**	*	*	*	*********
Pelà et al., 2022	Cohort	*	*	*	*	*		*	*	*******
Perlis et al., 2022	Cross-sectional	*	*			**				****
Loannou et al., 2022	Cohort	*	*	*	*	**	*	*	*	*********
Jacobs et al., 2023	Cross-sectional	*	*			**				****
Qasmieh et al., 2023	Cross-sectional	*	*			**				****
Quaranta et al., 2023	Cohort	*	*	*	*	**		*	*	********
Ramírez-Toscano et al., 2024	Cross-sectional	*	*	*		*				****
Resendez et al., 2024	Cohort	*	*	*	*	**	*	*	*	*********
Robertson et al., 2023	Cross-sectional	*	*			**				****
Rocha et al., 2024	Cohort	*	*	*	*	**		*	*	********
Romero-Rodríguez et al., 2023	Cross-sectional	*	*	*		**				*****
Wu et al., 2024	Cross-sectional	*	*			**				****
Shabnam et al., 2023	Cohort	*	*	*	*	**		*	*	********
Shigematsu et al., 2024	Cohort	*	*	*	*	**		*	*	********
Silva et al., 2023	Cohort	*	*	*		**		*	*	*******
Slurink et al., 2024	Cohort	*	*	*	*	**		*	*	********
Song and Giuriato, 2023	Cohort	*	*	*	*	**	*	*	*	*********
Stephens et al., 2024	Cohort	*	*	*	*	**	*	*	*	*********
Subramanian et al., 2022	Cohort	*	*	*	*	**	*	*	*	*********
Terai et al., 2023	Cohort	*	*	*	*	**	*	*	*	*********
Van Cleve et al., 2024	Cross-sectional	*	*			**				****
Wander et al., 2023	Cohort	*	*	*	*	**	*	*	*	*********
Wang et al., 2024	Cohort	*	*	*	*	**	*	*	*	*********
Wang et al., 2024	Cross -sectional	*	*	*		**				*****
Wilk et al., 2023	Cohort	*	*		*	**		*	*	*******
Wong et al., 2023	Cross -sectional	*	*			**				****
Pfaff et al., 2023	Cohort	*	*	*		**	*	*	*	********
Bai et al., 2022	Cohort	*	*	*	*	**	*	*	*	*********
Dryden et al., 2022	Cohort	*	*	*	*	**	*	*	*	*********
Asadi-Pooya et al., 2021	Cohort	*	*	*	*	**		*	*	********
Pazukhina et al., 2022	Cohort	*	*	*	*	**	*	*	*	*********
Moy et al., 2022	Cross-sectional	*				**				***
Khullar et al., 2023	Cohort	*	*	*	*	**	*	*	*	*********
Sharif-Askari et al., 2024	Cohort	*	*	*	*	**		*	*	********

Study quality was assessed using the Newcastle–Ottawa Scale (NOS), adapted for cohort and cross-sectional designs. Each asterisk (*) denotes one point per satisfied criterion; the Comparability domain allows up to two points (**). The total reflects points across Selection, Comparability, and Outcome domains

**Table 5:** Summary of quality assessment scores for studies included in the rapid review on social determinants of health and Long COVID

Study ID (Author, Year)	Study Design	Selection (0–4)	Comparability (0–2)	Outcome/Exposure (0–3)	Total Score (0–9)	Quality Rating (High, Medium, Low)
Adler et al., 2023	Cross-sectional	3	2	0	5	Moderate Quality
Atchison et al., 2023	Cross-sectional	2	2	0	5	Moderate Quality
Azambuja et al., 2024	Cohort	4	2	2	8	High Quality
Bonner et al., 2024	Cross-sectional	2	2	0	4	Moderate Quality
Bovil et al., 2022	Cross-sectional	2	2	0	4	Moderate Quality
Chelly et al., 2023	Cross-sectional	2	2	0	4	Moderate Quality
Chilunga et al., 2023	Cohort	4	2	3	9	High Quality
Chudzik et al., 2022	Cohort	3	2	2	7	High Quality
Coste et al., 2024	Cross-sectional	2	1	0	4	Moderate Quality
Crankson et al., 2022	Cross-sectional	3	2	0	5	Moderate Quality
Daniel et al., 2023	Cohort	4	2	2	8	High Quality
Durstenfeld et al., 2023	Cohort	2	2	1	5	Moderate Quality
Eligulashvili et al., 2024	Cohort	3	1	1	5	Moderate Quality
Feldstein et al., 2023	Cross-sectional	2	2	0	4	Moderate Quality
Ferreira et al., 2022	Cohort	4	2	3	9	High Quality
Feter et al., 2023	Cross-sectional	2	2	0	4	Moderate Quality
Evering et al., 2023	Cohort	4	2	1	7	High Quality
Fisher et al., 2024	Cross-sectional	2	2	0	4	Moderate Quality
Forster et al., 2022	Cohort	4	2	1	7	High Quality
Hejazian et al., 2024	Cross-sectional	2	2	1	5	Moderate Quality
Heller et al., 2022	Cross-sectional	2	2	0	4	Moderate Quality
Henderson et al., 2024	Cohort	4	2	3	9	High Quality
Hetlevik et al., 2023	Cohort	4	2	3	9	High Quality
Hossain et al., 2021	Cohort	4	2	2	8	High Quality
Krishnadath et al., 2023	Cohort	4	2	1	7	High Quality
Linh et al., 2024	Cross-sectional	3	2	0	5	Moderate Quality
Mahmoodi et al., 2023	Cross-sectional	2	2	0	4	Moderate Quality
Martinez-Ayala et al., 2023	Cohort	4	2	2	8	High Quality
Martin et al., 2024	Cross-sectional	2	2	0	4	Moderate Quality
Paranhos et al., 2022	Cross-sectional	3	2	1	6	Moderate Quality
Merzon et al., 2022	Cross-sectional	3	2	1	6	Moderate Quality
Miyazato et al., 2022	Cross-sectional	2	2	0	4	Moderate Quality
Mkoma et al., 2024	Cohort	4	2	3	9	High Quality
Modji et al., 2024	Cohort	4	2	2	8	High Quality
Mukherjee et al., 2022	Cohort	4	2	2	8	High Quality
Laughlin et al., 2023	Cohort	4	2	2	8	High Quality
Pastorello et al., 2025	Cohort	4	2	3	9	High Quality
Pelà et al., 2022	Cohort	4	1	2	7	High Quality
Perlis et al., 2022	Cross-sectional	2	2	0	4	Moderate Quality
Loannou et al., 2022	Cohort	4	2	2	8	High Quality
Jacobs et al., 2023	Cross-sectional	2	2	0	4	Moderate Quality
Qasmieh et al., 2023	Cross-sectional	2	2	0	4	Moderate Quality
Quaranta et al., 2023	Cohort	4	2	2	8	High Quality
Ramírez-Toscano et al., 2024	Cross-sectional	3	1	0	4	Moderate Quality
Resendez et al., 2024	Cohort	4	2	3	9	High Quality
Robertson et al., 2023	Cross-sectional	2	2	0	4	Moderate Quality
Rocha et al., 2024	Cohort	4	2	2	8	High Quality
Romero-Rodríguez et al., 2023	Cross-sectional	3	2	0	5	Moderate Quality
Wu et al., 2024	Cross-sectional	2	2	0	4	Moderate Quality
Shabnam et al., 2023	Cohort	4	2	2	8	High Quality
Shigematsu et al., 2024	Cohort	4	2	2	8	High Quality
Silva et al., 2023	Cohort	3	2	2	7	High Quality
Slurink et al., 2024	Cohort	4	2	2	8	High Quality
Song and Giuriato, 2023	Cohort	4	2	3	9	High Quality
Stephens et al., 2024	Cohort	4	2	3	9	High Quality
Subramanian et al., 2022	Cohort	4	2	3	9	High Quality
Terai et al., 2023	Cohort	4	2	3	9	High Quality
Van Cleve et al., 2024	Cross-sectional	2	2	0	4	Moderate Quality
Wander et al., 2023	Cohort	4	2	3	9	High Quality
Wang et al., 2024	Cohort	4	2	3	9	High Quality
Wang et al., 2024	Cross -sectional	3	2	0	5	Moderate Quality
Wilk et al., 2023	Cohort	3	2	2	7	High Quality
Wong et al., 2023	Cross -sectional	2	2	0	4	Moderate Quality
Pfaff et al., 2023	Cohort	3	2	3	8	High Quality
Bai et al., 2022	Cohort	4	2	3	9	High Quality
Dryden et al., 2022	Cohort	4	2	3	9	High Quality
Asadi-Pooya et al., 2021	Cohort	4	2	2	8	High Quality
Pazukhina et al., 2022	Cohort	4	2	3	9	High Quality
Moy et al., 2022	Cross-sectional	1	2	0	3	Low Quality
Khullar et al., 2023	Cohort	4	2	3	9	High Quality
Sharif-Askari et al., 2024	Cohort	4	2	2	8	High Quality

Quality scoring was performed using the Newcastle–Ottawa Scale (NOS). Scores span three domains—Selection (0–4), Comparability (0–2), and Outcome/Exposure (0–3)—with a total of 0–9. We categorized studies as High (7–9), Moderate (4–6), or Low (0–3) quality

**Table 6:** Risk of bias assessment for studies included in the rapid review on social determinants of health and Long COVID

Study	Design	Selection Bias	Comparability Bias	Outcome/Exposure Bias	Overall NOS Score	Overall Risk of Bias
Adler et al., 2023	Cross-sectional	Moderate (low 11.9% response rate and bias from parents selecting which child to report on)	Low (adjusted for age, sex, and infection status)	High (recall bias from parent-reported symptoms, no clinical validation)	5	Moderate
Atchison et al., 2023	Cross-sectional	Moderate (low 6.0% response rate and sample skewed toward older, white, affluent, and female participants)	Low (adjusted for age, sex, and SES factors)	High (recall bias from self-reported symptoms, proxy reporting for children, subjective outcomes like fatigue not validated clinically)	5	Moderate
Azambuja et al., 2024	Cohort	Low (community based recruitment with defined inclusion criteria, strong cohort representation for the study population)	Low (adjusted for age, sex, and SES factors)	Moderate (self-reported symptoms months after infection which may introduce recall bias)	8	Low
Bonner et al., 2024	Cross-sectional	Moderate (recruited via digital health platforms, may exclude individuals without internet access or lower digital literacy)	Low (adjusted for age, sex, and social vulnerability index)	High (recall bias from self-reported symptoms, no clinical assessment, or validated tools)	4	Moderate
Bovil et al., 2022	Cross-sectional	Moderate (non-random sampling from survey, limited to ≥ 50 years)	Low (adjusted for age, sex, education, comorbidity, hospitalization)	High (recall bias from self-reported symptoms, no duration criteria, no clinical validation)	4	Moderate
Chelly et al., 2023	Cross-sectional	Moderate (non-random sampling, limited representativeness)	Low (adjusted for age, sex, and symptoms)	High (recall bias from self-reported symptoms, no clinical verification)	4	Moderate
Chilunga et al., 2023	Cohort	Low (hospitalized population-based sample with exposed and comparison groups)	Low (adjusted for age, sex, comorbidities, and migration status)	Moderate (recall bias from self-reported symptoms by phone 3 months post-discharge, no clinical validation)	8	Low
Chudzik et al., 2022	Cohort	Moderate (PCR-positive patients from one hospital, may not reflect general population)	Low (adjusted for age, sex, and comorbidities)	Moderate (recall bias from self-reported symptoms without clinical confirmation)	7	Low
Coste et al., 2024	Cross-sectional	Moderate (random sampling, but only 55% response rate)	Moderate (adjusted for age and sex only)	High (recall bias from self-reported symptoms without clinical confirmation)	4	Moderate
Crankson et al., 2022	Cross-sectional	Moderate (hospital based sample with good sample size but limited to a single treatment center)	Low (adjusted for age, sex, education, and comorbidities)	High (long COVID outcomes based on retrospective self-report with no clinical confirmation, recall bias)	5	Moderate
Daniel et al., 2023	Cohort	Moderate (cohort from one health system, roughly 47% participation)	Low (adjusted for sociodemographics, illness severity, comorbidities)	Moderate (long COVID based on standardized phone interviews)	8	Low
Durstenfeld et al., 2023	Cohort	Moderate (Online recruitment may limit generalizability)	Low (adjusted for age, sex, variant wave, socioeconomic and medical history factors)	Moderate (Long COVID defined via self-reported symptom, recall bias)	5	Moderate
Eligulashvili et al., 2024	Cohort	Moderate (referral based, not population representative)	Moderate (adjusted for age and sex only)	High (self-reported symptoms, no clinical validation, recall bias)	5	Moderate
Feldstein et al., 2023	Cross-sectional	Moderate (survey based on previous respondents, may exclude those less likely to participate in online panels)	Low (adjusted for age, sex, education, marital status, health conditions, and vaccination status)	High (symptoms and test results self-reported, no clinical validation, recall bias)	4	Moderate
Ferreira et al., 2022	Cohort	Low (large hospital cohort, broad inclusion)	Low (adjusted for age, sex, comorbidities, SES)	Low (validated clinical tools, standardized tests, objective environmental exposure measures)	9	Low
Feter et al., 2023	Cross-sectional	Moderate (online convenience sampling via social media and networks)	Low (adjusted for sex, income, education, race, occupation, BMI, physical activity, chronic conditions, vaccination, hospitalization)	High (self-reported results and symptoms, no clinical validation, recall bias)	4	Moderate
Evering et al., 2023	Cohort	Low (randomized trial sample with strict inclusion criteria and diverse geographic sites)	Low (adjusted for age, sex, BMI, smoking, ethnicity, comorbidities, and baseline symptoms)	Moderate (self-reported symptoms and no clinical verification or controls, recall bias)	7	Low
Fisher et al., 2024	Cross-sectional	Moderate (online panel recruitment, lacks truly representative sampling frame)	Low (adjusted for age, sex, race/ethnicity, education, income, employment, insurance status)	High (self-report long COVID status, no clinical validation, recall bias)	4	Moderate
Forster et al., 2022	Cohort	Low (Total population sampling from PCR confirmed cases in 3 districts, high representativeness)	Low (adjusted for age, sex, comorbidity burden, hospitalization)	Moderate (Self-reported symptom, no clinical validation, recall bias)	7	Low
Hejazian et al., 2024	Cross-sectional	Moderate (45% response rate, excludes institutionalized and < 18 year old individuals)	Low (adjusted for age, sex, comorbidities, income, and health behaviors)	Moderate (self-reported symptoms without clinical verification, 3 months recall window which may may reduce recall bias)	5	Moderate
Heller et al., 2022	Cross-sectional	Moderate (quota sampling with low response rates, risk of underrepresentation of marginalized groups)	Low (adjusted for demographic, socioeconomic, and vaccination)	High (self-reported symptoms without clinical validation, recall bias)	4	Moderate
Henderson et al., 2024	Cohort	Low (very large, population based cohort of 19 million adults across England)	Low (adjusted for age, sex, NHS region, and dominant variant)	Low (outcomes based on standardized SNOMED codes in EHRs)	9	Low
Hetlevik et al., 2023	Cohort	Low (nationwide population based cohort with matched unexposed controls)	Low (adjusted for age, sex, symptoms, comorbidities)	Low (used standardized diagnostic codes over a fixed period, outcomes were objectively recorded and not self-reported).	9	Low
Hossain et al., 2021	Cohort	Low (Large cohort drawn from 24 testing facilities across Bangladesh)	Low (adjusted for age, sex, residence, comorbidities, occupation, smoking)	Moderate (symptoms self-reported during interviews, some objective cardiorespiratory measures used, no independent clinical validation)	8	Low
Krishnadath et al., 2023	Cohort	Low (population based recruitment from national COVID-19 database)	Low (adjusted for age, sex, ethnicity, and severity)	High (self-reported symptoms, only a small subset clinically examined, recall bias)	7	Low
Linh et al., 2024	Cross-sectional	Moderate (non-random sampling from a hospital list, exclusion of individuals with self-treated or mild cases limits generalizability)	Low (adjusted for sex, age group, BMI, vaccination status, and treatment duration)	High (self-reported symptoms without clinical confirmation, risk of recall bias from parent or child-reported data)	5	Moderate
Mahmoodi et al., 2023	Cross-sectional	Moderate (online survey, possible exclusion of low SES and older adults with limited internet access, non-random sample)	Low (adjusted for sex, education, reinfection, disease severity, and comorbidity)	High (self-reported symptom, no clinical validation, recall bias)	4	Moderate
Martínez-Ayala et al., 2023	Cohort	Low (large single center sample from confirmed PCR positive cases)	Low (adjusted for sex, COPD, rheumatic disease, mechanical ventilation, and hospitalization type)	Moderate (symptom persistence self-reported, no clinical validation, recall bias)	8	Low
Martin et al., 2024	Cross-sectional	Moderate (small sample size of 48 out of 207 contacted, and urban only population limit generalizability)	Low (adjusted for sex, income, and location)	High (self-reported SF-36 and PCFS scores with no clinical validation, recall bias)	4	Moderate
Paranhos et al., 2022	Cross-sectional	Moderate (sample recruited from a single rehab center in Brazil with exclusion of some patients, limiting generalizability, no random sampling)	Low (adjusted for age, sex, hospitalization, and multiple clinical symptoms including fatigue and anxiety)	High (olfactory dysfunction measured objectively, but other symptoms were self-reported, recall bias)	6	Moderate
Merzon et al., 2022	Cross-sectional	Moderate (population based sampling via health records, but diagnosis of Long COVID limited to coded records, may miss undiagnosed cases)	Low (adjusted for age, sex, SES, comorbidities including ADHD, urticaria, allergic rhinitis, etc.)	High (Long COVID diagnosis based on health record coding without clinical confirmation, possible misclassification or under coding)	6	Moderate
Miyazato et al., 2022	Cross-sectional	Moderate (survey limited to post-COVID plasmapheresis patients from a single center, and voluntary response)	Low (adjusted for age, sex, BMI, comorbidities)	High (self-reported symptoms with no clinical validation, timing of onset/recovery subject to recall bias)	4	Moderate
Mkoma et al., 2024	Cohort	Low (nationwide register based cohort of all COVID-19 positive adults in Denmark, highly representative of the population)	Low (adjusted for age, sex, civil status, education, income, and comorbidities)	Low (long COVID outcomes based on clinical ICD-10 diagnoses from national health registries, symptoms also captured via hospital records)	9	Low
Modji et al., 2024	Cohort	Low (statewide workers compensation dataset with linked records, excludes unreported claims)	Low (adjusted for age, sex, race, urbanicity, variant, vaccination)	Moderate (long COVID defined via ≥ 4 weeks of lost work time, lacks clinical validation)	8	Low
Mukherjee et al., 2022	Cohort	Moderate (based on commercial claims data; partial SDOH missing for 56%)	Low (adjusted for age, sex, race, income, education, comorbidities)	Moderate (ICD-10 code-based outcomes, lacks clinical validation)	8	Low
O’Laughlin et al., 2023	Cohort	Low (multicenter sample with clear inclusion/exclusion, strong sociodemographic diversity)	Low (adjusted for age, sex, SES, comorbidities, vaccination status, and SDOH)	Moderate (self-reported symptoms, activity levels, and missed work without clinical validation, recall bias)	8	Low
Pastorello et al., 2025	Cohort	Low (national random sample from tax database with weights to adjust for nonresponse, large representative cohort)	Low (adjusted for age, sex, SES, education, BMI, comorbidities, mental health, hospitalization, etc.)	Low (symptom timing aligned with acute episode, validated survey used, outcome based on standardized definition)	9	Low
Pelà et al., 2022	Cohort	Low (well defined cohort of 223 previously infected individuals, balanced enrollment of men and women)	Moderate (adjusted for age and sex only)	Moderate (Self-reported symptom, no clinical validation, recall bias)	8	Low
Perlis et al., 2022	Cross-sectional	Moderate (internet sample, preempaneled respondents, no reportable response rate, underrepresentation of those without internet access)	Low (adjusted for age, sex, education, income, urbanicity, vaccination, and variant exposure)	High (self-reported symptom, no clinical validation, recall bias)	4	Moderate
Ioannou et al., 2022	Cohort	Low (large national VA cohort with clear inclusion/exclusion criteria)	Low (adjusted for age, sex, race, SES, comorbidities)	Moderate (ICD 10 coded outcomes from EHR lack clinical validation, under detection of symptoms possible)	8	Low
Jacobs et al., 2023	Cross-sectional	Moderate (Survey had a large sample and stratified design, but potential nonresponse bias)	Low (Adjusted for age, sex, race, ethnicity, insurance, vaccination, region, household characteristics)	High (Self-reported long COVID and symptoms without clinical validation, recall bias)	4	Moderate
Qasmieh et al., 2023	Cross-sectional	Moderate (low response rate of 7.2%, reliance on opts in online panels)	Low (adjusted for age, sex, comorbidities, vaccination, race/ethnicity, education, income, employment, etc.)	High (self-reported infection, symptoms, with no clinical validation, recall bias)	4	Moderate
Quaranta et al., 2023	Cohort	Low (sampling of inpatients/outpatients with clear inclusion/exclusion criteria and broad representation of region)	Low (adjustment for age, sex, BMI, dyspnea, corticosteroid use etc.)	Moderate (self-reported symptoms without clinical validation, recall bias)	8	Low
Ramírez-Toscano et al., 2024	Cross-sectional	Moderate (probabilistic stratified sample with 72% participation, but proxy reporting by household heads)	Moderate (adjusted for sex and age)	High (recall bias from self-reported symptoms without clinical confirmation)	4	Moderate
Resendez et al., 2024	Cohort	Low (large national VA sample with, robust inclusion criteria, before-after crossover design using patients as their own controls)	Low (adjusted for age, sex, race, Elixhauser score, vaccination status, and oxygen saturation)	Low (ICD-10-CM diagnoses extracted from EHRs, novel diagnoses tracked, objective outcomes with high specificity)	9	Low
Robertson et al., 2023	Cross-sectional	Moderate (multi mode survey with weighting, but partial online opt in panel)	Low (adjusted for age, sex, comorbidities, vaccination, income, and insurance)	Moderate (Self reported symptom, no clinical validation, recall bias)	4	Moderate
Rocha et al., 2024	Cohort	Moderate (convenience sample from 3 of 7 hospitals, exclusions for communication barriers)	Low (adjusted for age, sex, income, comorbidities)	Moderate (self-reported symptoms, no clinical validation, recall bias)	8	Low
Romero-Rodríguez et al., 2023	Cross-sectional	Moderate (self-selected participants via online survey; limited to Spanish primary care professionals, mostly urban)	Low (adjusted for age, sex, and several comorbidities)	High (all symptom and exposure data were self-reported with no clinical confirmation)	5	Moderate
Wu et al., 2024	Cross-sectional	Moderate (population based national surveys, underrepresentation due to nonresponse and exclusion of individuals w/o responses)	Low (adjusted for as age, sex, race/ethnicity, education, urbanicity, and COVID severity)	High (self-reported symptoms, no clinical validation, no information on symptom duration beyond 3-month threshold)	4	Moderate
Shabnam et al., 2023	Cohort	Low (nationally representative sample using random household selection and broad inclusion of SARS-CoV-2-infected individuals)	Low (adjusted for age, sex, ethnicity, urban/rural location, etc.)	Moderate (symptoms were self-reported without clinical validation, recall bias)	8	Low
Shigematsu et al., 2024	Cohort	Low (multi-center prospective cohort across 26 hospitals in Japan)	Low (adjusted for age, sex, BMI, asthma etc.)	Moderate (brain fog defined via self-reported symptoms of memory impairment or poor concentration without clinical testing, recall bias)	8	Low
Silva et al., 2023	Cohort	Moderate (Prospective design with large sample, sample derived from reachable individuals via phone follow up only)	Low (adjusted for age, sex, race, acute symptoms, BMI, and reinfection)	Moderate (Outcomes were self-reported without clinical validation, recall bias, reinfection timing may have overlapped with symptom assessment for some participants)	7	Low
Slurink et al., 2024	Cohort	Low (probability-based national panel with high 79.5% response rate; representative of the Dutch population)	Low (adjusted for age, sex, ethnicity, educational level, marital status, household income, BMI etc.)	Moderate (self-reported symptoms without clinical verification, recall bias, but mitigated by panel design and pre pandemic baseline data)	8	Low
Song and Giuriato, 2023	Cohort	Low (nationwide insured sample, continuous enrollment, defined comparison groups)	Low (adjusted for age, sex, and month-year of infection; Elixhauser Index)	Low (claims-based outcomes using standardized ICD-10 codes and symptom criteria)	9	Low
Stephens et al., 2024	Cohort	Low (Large, national VA cohort)	Low (adjusted for age, sex, race/ethnicity, education, income, household size, rural/urban residence etc.)	Low (ICD-10 diagnoses and electronic medical record symptoms, objective data from national VA records)	9	Low
Subramanian et al., 2022	Cohort	Low (large representative UK primary care dataset with propensity score matching)	Low (adjusted for age, sex, ethnicity, socioeconomic status, BMI, smoking status, comorbidities etc.)	Low (symptoms drawn from structured clinical records with 115 symptoms assessed)	9	Low
Terai et al., 2023	Cohort	Low (nationwide multicenter prospective cohort, large sample size, inpatient population representative)	Low (adjusted for age, sex, severity, smoking, etc.)	Low (symptoms tracked at multiple time points using standardized tools, high patient follow-up)	9	Low
Van Cleve et al., 2024	Cross-sectional	Moderate (veteran only panel, limiting generalizability)	Low (adjusted for age, sex, education, income, etc.)	High (self-reported symptoms, no clinical validation, recall bias)	4	Moderate
Wander et al., 2023	Cohort	Low (large national VA sample with defined inclusion)	Low (adjusted for age, sex, race/ethnicity etc.)	Low (EHR and claims based U09.9 code, medical record validation)	9	Low
Wang et al., 2024	Cohort	Low (large, representative EHR sample)	Low (adjusted for age, sex, ethnicity, BMI, deprivation, etc.)	Low (Clinically confirmed diagnosis and symptom coding from EHRs)	9	Low
Wang et al., 2024	Cross-sectional	Moderate (community based recruitment via public health institutions, but limited to older adults)	Low (adjusted for age, sex, education, marital status, ethnicity etc.)	High (recall bias from self-reported symptoms, no clinical validation or objective measurement, subjective outcomes)	5	Moderate
Wilk et al., 2023	Cohort	Moderate (Limited to ≥ 50 adults, based on respondents to prior SHARE surveys who opted into follow up)	Low (adjusted for age, sex, education, employment, multimorbidity, immigration, and income)	Moderate (self-reported symptoms, no clinical validation, recall bias)	7	Low
Wong et al., 2023	Cross-sectional	Moderate (online Qualtrics survey; underrepresentation of older adults, survey restricted to digital device users)	Low (adjusted for age, sex, occupation, income, education, severity etc.)	High (self-reported symptoms, no clinical validation, recall bias)	4	Moderate
Pfaff et al., 2023	Cohort	Low (large multi-site retrospective cohort from EHRs, inclusion criteria clearly defined, representative of long COVID patients)	Low (adjusted for age, sex, poverty, unemployment, insurance coverage, and education level)	Low (HER based data including diagnosis codes, procedures, and medication use, outcome timing clearly defined)	8	Low
Bai et al., 2022	Cohort	Low (single centre study with follow-up of 77% of discharged patients, clear inclusion/exclusion criteria)	Low (adjusted for age, sex, severity of illness, comorbidities, BMI etc.)	Moderate (self-reported symptoms, validated tools used for psychological symptoms, but no clinical verification of physical symptoms, recall bias)	9	Low
Dryden et al., 2022	Cohort	Low (large, nationally representative cohort using random sampling from hospital admissions with high response rate)	Low (adjusted for age, sex, ethnicity, comorbidities etc.)	Low (used structured questionnaires and tools, followed patients over time)	9	Low
Asadi-Pooya et al., 2021	Cohort	Low (large population-based sample, broad inclusion across 55 centers)	Low (adjusted for age, sex, respiratory symptoms at onset, ICU admission, hospital stay length)	Moderate (symptoms self-reported by phone, no clinical validation or control group, recall bias)	8	Low
Pazukhina et al., 2022	Cohort	Low (PCR confirmed hospitalized patients from multiple hospitals, strong cohort definition)	Low (adjusted for age, sex, comorbidities, severity)	Low (used consistent symptom checklists, with follow-up at 6 and 12 months)	9	Low
Moy et al., 2022	Cross-sectional	High (online convenience sample, skewed toward younger, urban, and educated respondents; under representation of elderly)	Low (adjusted for sex, comorbidities, BMI, severity)	High (self-reported symptoms, no clinical validation or symptom duration requirement, recall bias)	3	High
Khullar et al., 2023	Cohort	Low (large EHR-based sample across 5 NYC systems)	Low (adjusted for age, sex, time, comorbidities etc.)	Low (outcomes from clinician entered ICD-10 codes with structured follow up period)	9	Low
Sharif-Askari et al., 2024	Cohort	Low (large group of non-hospitalized adults from clinics across Dubai, using electronic health records)	Low (adjusted for age, sex, race, BMI etc.)	Moderate (long COVID based on physician coded visits, no symptom severity scale)	8	Low

We adapted the Newcastle–Ottawa Scale to evaluate Selection, Comparability, and Outcome/Exposure domains. The Overall NOS Score ranges from 0 to 9; higher scores indicate lower risk of bias. We categorized overall risk as: Low risk (7–9), Moderate risk (4–6), and High risk (0–3)

Abbreviation: *BMI* Body mass index, *COPD* Chronic obstructive pulmonary disease, *EHR* Electronic health record, *ICD-10/ICD-10-CM* International classification of diseases, 10th revision (Clinical modification), *ICU* Intensive care unit, *NHS* National health service, *NOS* Newcastle–ottawa scale, *PCR* Polymerase chain reaction, *PCFS* Post-COVID-19 functional status, *SF-36* Short form health survey, *SNOMED* Systematized nomenclature of medicine, *SVI* Social vulnerability index, *VA* Veterans affairs

Limitations were most observed in the outcome/exposure domain, where 65% of studies (*n* = 46) scored below the maximum of three out of three. These limitations were primarily due to reliance on self-reported symptoms, lack of clinical validation, or inadequate follow-up, particularly in cross-sectional studies. In the selection domain, 49% of studies (*n* = 35) scored below four out of four, often reflecting sampling issues or lack of clarity in how exposures were defined or measured. In contrast, 94% (*n* = 67) performed well in the comparability domain, scoring two out of two by adjusting for key confounders such as age and sex, as well as additional relevant factors. The single low-quality study scored zero out of three in the outcome/exposure domain, one out of four in selection, and two out of two in comparability, reflecting substantial risk of measurement and selection bias.

## Discussion

The rapid review highlights the multifaceted impact of social determinants of health on Long COVID. Consistent themes included higher risk among females [24, 25, 27–36, 38, 40, 42, 43, 46, 47, 49, 51, 52, 54–58, 60–64, 66, 67, 69, 72–77, 81, 85, 89, 90, 92, 94, 95] and older adults [30, 34, 35, 37, 38, 41, 43, 44, 49, 50, 54, 55, 71, 72, 74, 76, 90, 91], disparities across racial [36, 37, 75, 83] and ethnic [34, 37, 48, 83] groups, and increased vulnerability linked to socioeconomic disadvantage [26, 36, 42, 58, 70, 73, 74, 84–86]. Geographical location [34, 38, 45, 50, 53, 74, 76, 80, 83] and occupation [42, 50, 53, 69] also emerged as important but context-dependent factors. These patterns highlight that Long COVID is not only a clinical condition, but a population health issue shaped by the distribution of social and structural determinants across communities. Understanding these factors is essential for developing equitable prevention and management strategies at the health system and policy levels.

The findings of this rapid review align with and expand upon existing literature examining the role of SDoH and Long COVID. The results demonstrate that females are more likely to develop Long COVID, aligning with previous reviews that identify female sex as a risk factor for Long COVID [95–102]. A systematic review and meta-analysis synthesized data from 41 studies, including 860,783 patients, found that female sex was significantly associated with an increased risk of developing Long COVID (OR 1.56, 95% CI 1.41–1.73) [100]. A systematic review and meta-analysis of pediatric populations, including 16 observational studies with 46,262 participants, found low certainty evidence suggesting that female sex may be associated with an increased risk of Long COVID in children and adolescents [102]. 

Hormonal differences, may contribute to prolonged inflammation following SARS-CoV-2 infection, sustaining a hyperinflammatory state even after recovery. Females produce stronger IgG antibody responses during the acute phase of COVID-19, which may lead to prolonged immune activation and symptom persistence [100]. 

Despite these findings, one study found that women were less likely to be diagnosed with Long COVID than men [88]. This discrepancy between symptom burden and diagnosis may reflect gender differences in healthcare-seeking behaviors, clinician recognition and coding practices, or systemic biases in how women’s symptoms are evaluated in clinical settings. Future research should investigate these dynamics to clarify whether disparities arise from biological differences, healthcare access, or structural biases in diagnosis and reporting.

Our findings also showed that older individuals are at greater risk for developing Long COVID, a trend that has been widely documented in previous reviews and meta-analyses [95–102]. A systematic review and meta-analysis found that adults aged over 40 were at higher risk of developing Long COVID compared to younger adults (OR 1.21, 95% CI 1.11–1.33) [100], with similar patterns observed in pediatric populations where increasing age correlates to increased risk (OR 1.30, 95% CI 1.15–1.46) [102]. This is likely due to older adults generally experiencing more severe cases of acute COVID-19, resulting in increased organ damage and lasting effects like persistent fatigue and reduced lung function, all of which indicate reduced organ performance and a slower recovery process [95]. The higher prevalence of pre-existing conditions and comorbidities among older adults may further contribute to their increased vulnerability [50]. For example, patients aged 65 and older often have chronic conditions like congestive heart failure alongside Long COVID symptoms. These comorbidities likely reflect aging rather than Long COVID symptoms, as their incidence increases with age, thereby complicating diagnosis [31]. Furthermore, because Long COVID is diagnosed in those who survive the acute phase of infection, its prevalence may be underestimated in older populations, as those with multiple comorbidities face a higher risk of severe illness and mortality before reaching the stage where Long COVID symptoms appear [100]. 

In terms of race/ethnicity, our findings align with previous reviews where Black and Hispanic populations face an increased risk of Long COVID [98, 99]. A previous review highlighted that Black and Hispanic individuals experience greater prevalence and burden of Long COVID, with reduced healthcare access and higher occupational exposures frequently identified as contributing factors [98]. Additional mechanisms include poor housing conditions, financial instability, and limited access to healthcare, all of which increase vulnerability to severe COVID-19 and its long-term sequelae [98]. 

In contract, a systematic review and meta-analysis of pediatric populations (16 observational studies; 46,262 participants) found that Asian and Black individuals may have a lower risk of developing Long COVID (OR 0.62, 95% CI 0.45–0.85), though the certainty of this evidence was low [102]. Similarly, one review reported that non-Black individuals had a higher likelihood of Long COVID [96], while another found that studies with a higher proportion of non-White participants reported greater prevalence of Long COVID [97]. These discrepancies likely reflect differences in study design, populations, and diagnostic approaches. Definitions of Long COVID ranged from self-reported symptoms to administrative codes, with variability in follow-up duration, patient demographics, and baseline health status. Such heterogeneity complicates cross-study comparisons and underscores the need for standardized methods to better understand racial and ethnic disparities in Long COVID.

In addition, this review supports an association between lower educational attainment and increased risk of Long COVID. This finding is consistent with a previous review that reported higher prevalence among individuals with lower education levels in Africa [96]. One possible explanation is that limited health literacy may make it more challenging to differentiate between symptoms of pre-existing chronic conditions and those attributable to COVID [91], which could impact how symptoms are reported. At the same time, individuals with higher educational attainment may be more likely to engage in health-seeking behaviors and have better access to timely and appropriate healthcare services, which could contribute to differences in diagnosis and management.

Our findings suggest that lower financial security is generally associated with increased risk of Long COVID, though evidence is not entirely consistent. Some studies reported that financial instability was linked to worse outcomes [69], while others found no direct association [98], and one study even observed a lower likelihood of Long COVID in the most deprived areas [33].

Potential pathways include higher prevalence of comorbidities [70], limited access to healthcare, and greater exposure to infection due to factors such as overcrowded housing and shared living spaces [69]. These conditions not only elevate the risk of acquiring COVID-19 but may also contribute to prolonged recovery and greater symptom burden.

To our knowledge, this is the first review to examine the role of occupation in Long COVID risk. We found that individuals in frontline and public-facing roles appear to face an elevated risk, likely reflecting greater exposure to SARS-CoV-2 and subsequent susceptibility to persistent symptoms [69]. This represents an important and novel contribution to the literature, as occupational exposures have been underexplored despite their clear relevance for both risk stratification and workplace policy.

Similarly, no prior reviews have assessed the role of geographical area in Long COVID risk. Of the 19 studies assessing this relationship, just over half reported significant associations [34, 37, 38, 45, 50, 53, 74, 76, 80, 83], though the direction and magnitude of effects varied. Rural residence was linked to higher prevalence in some studies [53, 74], while others found elevated risk among urban veterans [37], metropolitan workers [50], or southern U.S. residents [83]. At the same time, several studies reported urban residence as protective or associated with lower symptom prevalence. Differences also appeared in healthcare use and documentation, with urban residents more likely to seek care [45] but less likely to have Long COVID formally coded. These inconsistent results suggest that geographic factors could impact both risk and recognition of Long COVID, though variations in study design, population, healthcare systems, vaccination policies, and testing rates likely contribute to the heterogeneity.

Likewise, employment status has not been examined in previous review, yet our findings indicate that unemployed individuals face a higher risk of developing Long COVID. While the studies reporting this association did not provide explicit explanations, potential contributing factors include greater barriers to healthcare access and higher levels of psychosocial stress, such as financial strain, which may increase vulnerability to persistent symptoms after acute SARS-CoV-2 infection.

### Population health and policy implications

The synthesis of evidence across diverse contexts highlights that Long COVID must be addressed through coordinated public health and policy responses, not only through clinical care. Policymakers and public health agencies can use these insights to identify groups at greatest risk and guide the equitable allocation of resources for rehabilitation and follow-up care [103]. Integrating Long COVID surveillance into existing chronic disease monitoring systems would help track long-term outcomes across socioeconomic and geographic contexts. At the same time, employment protection, income support, and accessible sick-leave policies can reduce financial strain and enable recovery, particularly for workers in public-facing or insecure jobs. In healthcare systems, embedding screening for social needs within primary care and post-COVID care centers could help link patients to community supports, improving recovery trajectories and reducing inequities in long-term outcomes.

### Strengths and limitations

This review synthesizes evidence from 71 studies across multiple countries, providing one of the most comprehensive evaluations to date of the relationship between social determinants of health and Long COVID. A key strength is the inclusion of geographically diverse studies, which enhances the generalizability of findings across different populations and healthcare systems. The review also employed a comprehensive, peer-reviewed search strategy, across multiple databases, capturing a wide range of evidence and addressing gaps in prior reviews, such as the limited consideration of occupation and employment status as well as geographical areas. Importantly, this review underscores the need for standardized definitions of Long COVID, as variation in case definitions remains a critical barrier to comparability and should be a key priority for future research.

Nonetheless, several limitations must be acknowledged. Definition of Long COVID varied across studies with inconsistent duration thresholds contributing to differences in reported prevalence. The reliance on observational designs limits causal inference, as unmeasured confounding and residual bias may have influenced observed associations between social determinants and Long COVID. The exclusion of qualitative research further limits understanding of lived experiences and contextual factors. In some studies, reliance on self-reported data may have introduced recall bias, and short follow-up periods could underestimate symptom burden and persistence. Restricting the search to English-language publications may also have excluded relevant studies conducted in other contexts. Future work should not only standardize definitions and outcome measures but also incorporate mixed-methods approaches to better capture the intersectionality of social determinants and the lived experience of Long COVID.

### Future directions

The findings of this review call for the urgent need of equitable healthcare access, targeted public health interventions, and standardized research frameworks to address disparities in Long COVID outcomes.

The first step should be expanding research to include more pediatric populations. Most Long COVID research has focused on adults, leaving significant gaps in understanding its impact on children and adolescents. This will help identify any age specific risk factors, symptom patterns and effective treatment strategies. Since children may experience unique long-term effects, research should prioritize early detection and in younger age groups. Secondly, it is important to standardize definitions of Long COVID across all studies as the way it is defined and measured affects prevalence estimates. Variations in how Long COVID is defined and diagnosed make it difficult to compare studies and develop effective treatments. Establishing a standardized definition across different healthcare systems and research settings will improve consistency in data collection and enhance comparability of findings. Lastly, exploring the interaction of multiple social determinants is important. Long COVID does not affect all populations equally, and overlapping vulnerabilities can compound risks. Collectively, these findings call for a coordinated population health response that bridges clinical, public health, and social policy domains. Future research should evaluate policy and system-level interventions that address these inequities. For example, programs that integrate social prescribing, occupational health supports, and post-COVID rehabilitation within primary care. Establishing standardized data collection on social determinants in Long COVID cohorts will also be critical for informing population-level planning and preparedness for future outbreaks.

## Conclusion

This review emphasizes the role that social determinants of health play in shaping the risk and severity of Long COVID. Across 71 studies, evidence frequently showed that demographic factors (female sex, older age), socioeconomic disadvantage (lower education, financial insecurity, unemployment), occupational exposures (frontline and essential work), and geographic context contribute to unequal outcomes. These findings confirm that Long COVID is not experienced equally across populations. Addressing these disparities will require targeted public health efforts, including equitable access to healthcare, tailored outreach to vulnerable groups, and stronger occupational protections for high-risk workers. Standardized case definitions remain essential for consistent diagnosis and cross-study comparability. Future research should prioritize pediatric populations, the intersection of multiple social determinants of health, and longitudinal designs to better assess persistence, recovery trajectories, and the long-term impact of Long COVID.

## Supplementary Information

Additional file 1. Detailed database search strategies and applied filters used to identify studies examining the relationship between social determinants of health and Long COVID.

Additional file 2. Completed PRISMA 2020 checklist documenting compliance with preferred reporting items for systematic reviews.

Additional file 3. Summary of study design, population, geographic location, definitions of Long COVID, social determinants examined, and key findings extracted from each included study.

## Data Availability

The datasets used and/or analysed during the current study are available from the corresponding author upon reasonable request.
