# Supplementary material for: Understanding how social determinants of health shape Long COVID outcomes: a rapid review of evidence
Source: Arch Public Health. 2025 Dec 29;83:308. doi: 10.1186/s13690-025-01787-x (PMC12751510; doi:10.1186/s13690-025-01787-x)
Supplement: Supplementary file 1 — Additional file 1. Detailed database search strategies and applied filters used to identify studies examining the relationship between social determinants of health and Long COVID. [file 13690_2025_1787_MOESM1_ESM.pdf]

Additional File 1. Detailed database search strategies and applied filters used to identify studies examining the relationship between social determinants of health and Long COVID

Social Determinants of Health – Long COVID
Final Strategies
2024 Sep 29

Ovid Multfile

Database: Embase Classic+Embase <1947 to 2024 September 27>, Ovid MEDLINE(R) ALL <1946 to September 26, 2024>
Search Strategy:

----

1 Post-Acute COVID-19 Syndrome/ (8220)
2 ((long* or post*) adj (COVID or COVID-19 or COVID19 or coronavirus* or corona virus*)).tw,kw,kf. (44431)
3 ((long-term or longterm or post-acute or postacute or chronic) adj3 (COVID or COVID-19 or COVID19 or coronavirus* or corona virus* or 2019-nCoV or 19nCoV or 2019nCoV or SARS-CoV-2 or SARS-CoV2 or SARSCoV-2 or SARSCoV2 or SARS2 or SARS-2 or severe acute respiratory syndrome coronavirus 2)).tw,kw,kf. (12987)
4 (("long* haul*" or longhaul* or "long* tail*" or longtail* or longduration* or "long duration*" or longlast* or "long last*" or longstanding* or "long standing*" or "medium* term*" or mediumterm*) adj3 (COVID or COVID-19 or COVID19 or coronavirus* or corona virus* or 2019-nCoV or 19nCoV or 2019nCoV or SARS-CoV-2 or SARS-CoV2 or SARSCoV-2 or SARSCoV2 or SARS2 or SARS-2 or severe acute respiratory syndrome coronavirus 2)).tw,kw,kf. (1169)
5 ((nonrecover* or "non recover*" or "not recover*") adj3 (COVID or COVID-19 or COVID19 or coronavirus* or corona virus* or 2019-nCoV or 19nCoV or 2019nCoV or SARS-CoV-2 or SARS-CoV2 or SARSCoV-2 or SARSCoV2 or SARS2 or SARS-2 or severe acute respiratory syndrome coronavirus 2)).tw,kw,kf. (47)
6 ((after discharg* or following discharg* or postacute* or "post acute*" or postdischarg* or "post discharge" or "post discharging" or posthospital* or post-hospital* or postinfect* or "post infection" or "post infective*" or postviral* or "post viral*" or postvirus* or "post virus*" or postcritical or post-critical or postintensive or post-intensive or post-ICU) adj3 (complication? or consequence? or convalescen* or disabilit* or feature* or illness* or prognos* or sequela* or sign or signs or suffering? or symptom* or recuperat*) adj3 (COVID or COVID-19 or COVID19 or coronavirus* or corona virus* or 2019-nCoV or 19nCoV or 2019nCoV or SARS-CoV-2 or SARS-CoV2 or SARSCoV-2 or SARSCoV2 or SARS2 or SARS-2 or severe acute respiratory syndrome coronavirus 2)).tw,kw,kf. (3252)
7 ((chronic* or continuous* or continual* or continuing* or delay* or endur* or extend* or fluctuat* or gradual* or lasting* or legacy* or lengthy* or linger* or long* or "medium* term*" or mediumterm* or multisystem* or "multi system*" or ongoing or permanent* or persist* or prolong* or protract* or relaps* or remission* or remit* or residual* or slow* or subacute* or "sub acute*") adj3 (complication? or consequence? or convalescen* or disabilit* or feature* or illness* or prognos* or sequela* or sign or signs or suffering? or symptom* or recuperat*) adj3 (COVID or COVID-19 or COVID19 or coronavirus* or corona virus* or 2019-nCoV or 19nCoV or 2019nCoV or SARS-CoV-2 or SARS-CoV2 or SARSCoV-2 or SARSCoV2 or SARS2 or SARS-2 or severe acute respiratory syndrome coronavirus 2)).tw,kw,kf. (8713)

8 ((after discharg* or following discharg* or postacute* or "post acute*" or postdischarg* or "post discharge" or "post discharging" or posthospital* or post-hospital* or postinfect* or "post infection" or "post infective*" or postviral* or "post viral*" or postvirus* or "post virus*" or postcritical or post-critical or postintensive or post-intensive or post-ICU) adj3 recover* adj3 (COVID or COVID-19 or COVID19 or coronavirus* or corona virus* or 2019-nCoV or 19nCoV or 2019nCoV or SARS-CoV-2 or SARS-CoV2 or SARSCoV-2 or SARSCoV2 or SARS2 or SARS-2 or severe acute respiratory syndrome coronavirus 2)).tw,kw,kf. (104)
9 ((chronic* or continuous* or continual* or continuing* or delay* or endur* or extend* or fluctuat* or gradual* or lasting* or legacy* or lengthy* or linger* or long* or "medium* term*" or mediumterm* or multisystem* or "multi system*" or ongoing* or permanent* or persist* or prolong* or protract* or relaps* or remission* or remit* or residual* or slow* or subacute* or "sub acute*") adj3 recover* adj3 (COVID or COVID-19 or COVID19 or coronavirus* or corona virus* or 2019-nCoV or 19nCoV or 2019nCoV or SARS-CoV-2 or SARS-CoV2 or SARSCoV-2 or SARSCoV2 or SARS2 or SARS-2 or severe acute respiratory syndrome coronavirus 2)).tw,kw,kf. (706)
10 (following adj (COVID or COVID-19 or COVID19 or coronavirus* or corona virus* or 2019-nCoV or 19nCoV or 2019nCoV or SARS-CoV-2 or SARS-CoV2 or SARSCoV-2 or SARSCoV2 or SARS2 or SARS-2 or severe acute respiratory syndrome coronavirus 2) adj3 (comorbid* or "co morbid*" or condition* or convalescen* or disease* or disorder* or illness* or multimorbid* or "multi morbid*" or sickness* or symptom* or syndrome* or sign or signs or prognos* or recuperat* or survivor* or survival* or risk*)).tw,kw,kf. (2471)
11 (after adj (COVID or COVID-19 or COVID19 or coronavirus* or corona virus* or 2019-nCoV or 19nCoV or 2019nCoV or SARS-CoV-2 or SARS-CoV2 or SARSCoV-2 or SARSCoV2 or SARS2 or SARS-2 or severe acute respiratory syndrome coronavirus 2) adj3 (comorbid* or "co morbid*" or condition* or convalescen* or disease* or disorder* or illness* or multimorbid* or "multi morbid*" or sickness* or symptom* or syndrome* or sign or signs or prognos* or recuperat* or survivor* or survival* or risk*)).tw,kw,kf. (4418)
12 or/1-11 [LONG COVID PT 1] (58633)
13 COVID-19/ (438515)
14 (COVID or COVID-19 or COVID19 or coronavirus* or corona virus* or 2019-nCoV or 19nCoV or 2019nCoV or SARS-CoV-2 or SARS-CoV2 or SARSCoV-2 or SARSCoV2 or SARS2 or SARS-2 or severe acute respiratory syndrome coronavirus 2).ti,kw,kf. (837927)
15 or/13-14 (905002)
16 Long Term Adverse Effects/ (262453)
17 "Recovery of Function"/ (121237)
18 Convalescence/ (69581)
19 or/16-18 (392726)
20 15 and 19 [LONG COVID PT 2] (8338)
21 12 or 20 [LONG COVID PTS 1-2] (65852)
22 "Social Determinants of Health"/ (33577)
23 ((social* or socioeconomic* or socio-economic*) adj determin*).tw,kw,kf. (51239)
24 ((commercial* or structural*) adj3 determin* adj3 health*).tw,kw,kf. (2003)
25 ((nonmedical or non-medical) adj (factor? or variables)).tw,kw,kf. (1170)
26 Sociological Factors/ (1875)
27 ((social or sociologic*) adj3 (arrangement? or class$2 or condition? or environment? or gradient? or situation?)).tw,kw,kf. (101440)
28 ((social or sociologic*) adj3 (aspect? or caus* or component? or consider* or detail? or effect? or element* or facet? or factor? or feature? or influen* or patient or patients or reason* or variables)).tw,kw,kf. (254356)

29 Social Conditions/ (139247)
30 ((accommodat* or housing or home? or living) adj3 (arrangement? or condition? or environment? or factor? or situation? or variables)).tw,kw,kf. (113799)
31 ((accommodat* or housing or home? or living) adj3 (access* or availabl* or insecur* or secur* or instabilit* or stabilit* or stable or unstable or precarious* or marginal* or safe or safety or unsafe or vulnerab*)).tw,kw,kf. (38541)
32 Food Security/ (11494)
33 ((food or grocer* or nutritio*) adj3 (access* or availab* or insecur* or secur* or instabilit* or stabilit* or stable or unstable or suppli* or supply* or precarious* or marginal* or safe or safety or unsafe or vulnerab*)).tw,kw,kf. (172483)
34 ((exercis* or physical activit* or sport*) adj3 (access* or availab* or insecur* or secur* or instabilit* or stabilit* or stable or unstable or suppli* or supply* or opportunit* or precarious* or marginal* or safe or safety or unsafe)).tw,kw,kf. (23159)
35 (amenity or amenities).tw,kw,kf. (4255)
36 Employment/ (140010)
37 Unemployment/ (38181)
38 Job Security/ (1333)
39 ((employ* or job or jobs or work*) adj3 (abilit* or access* or availab* or insecur* or secur* or instabilit* or stabilit* or stable or unstable or condition? or opportunit* or precarious* or marginal* or safe or safety or unsafe or vulnerab*)).tw,kw,kf. (205916)
40 unemploy*.tw,kw,kf. (58842)
41 Sociodemographic Factors/ (27757)
42 ((sociodemograph* or socio-demograph*) adj3 (aspect? or caus* or component? or consider* or detail? or effect? or element* or facet? or factor? or feature? or influen* or patient or patients or reason* or variables)).tw,kw,kf. (111759)
43 exp Socioeconomic Factors/ (2154106)
44 ((socioeconomic* or socio-economic*) adj3 (aspect? or caus* or component? or consider* or detail? or effect? or element* or facet? or factor? or feature? or influen* or patient or patients or reason* or variables)).tw,kw,kf. (107229)
45 Economic Stability/ (402)
46 ((economic* or financ* or income*) adj3 (insecur* or secur* or instabilit* or stabilit* or stable or unstable or vulnerab*)).tw,kw,kf. (22863)
47 (economic* adj3 (condition? or environment? or factor? or situation? or variables)).tw,kw,kf. (78734)
48 Income/ (111478)
49 (poverty or impoverish* or limited income? or low* income? or reduced income?).tw,kw,kf. (222047)
50 Education/ (558065)
51 Educational Status/ (185353)
52 ((academic* or education* or highschool* or high-school* or school*) adj3 (abilit* or achiev* or attain* or background* or enroll* or failure? or graduat* or level? or quality or status or success*)).tw,kw,kf. (434887)
53 Literacy/ (9563)
54 (literate or literac* or illiterate* or illiterac*).tw,kw,kf. (97877)
55 ((read* or writ*) adj3 (abilit* or limit* or proficienc* or skill*)).tw,kw,kf. (28938)
56 ((language? or speak*) adj3 (abilit* or limit* or proficienc* or skill*)).tw,kw,kf. (39762)
57 Limited English Proficiency/ (1205)
58 Digital Divide/ (1136)

59 (digital adj (divide or division?)).tw,kw,kf. (2862)
60 ((earl* child* or earl* year?) adj3 (develop* or educat*)).tw,kw,kf. (12151)
61 exp Health Services Accessibility/ (270191)
62 ((care or healthcare or health care or health service?) adj3 (access* or availab* or equit*)).tw,kw,kf. (215655)
63 Health Literacy/ (33360)
64 ((health or healthcare) adj3 (literac* or literate)).tw,kw,kf. (42035)
65 Prejudice/ (30417)
66 (discriminat* or prejudic*).tw,kw,kf. (732323)
67 (antisemit* or anti-semit* or islamophobi* or islamo-phobi*).tw,kw,kf. (919)
68 Xenophobia/ (548)
69 (xenophobi* or xeno-phobi*).tw,kw,kf. (1049)
70 Incarceration/ (2276)
71 Prisoners/ (39202)
72 (incarcerat* or prison* or imprison* or jail*).tw,kw,kf. (85614)
73 exp Ethnic Groups/ (318522)
74 exp Ethnicity/ (247815)
75 ((ethnic* or nationalit*) adj3 (access* or balanc* or bias* or capacity-building or discrepanc* or discriminat* or disparit* or divers* or equal* or equit* or gap or gaps or harass* or identit* or imbalanc* or inclusi* or inequalit* or in-equal* or inequit* or in-equit* or objectif* or parity or prejudic* or recruit* or represent* or status* or stereotyp* or stereo-typ* or stigma* or underrepresent* or under-represent* or unequal* or un-equal* or vulnerab*)).tw,kw,kf. (96964)
76 ethnic*.tw,kw,kf. (492505)
77 exp "Emigrants and Immigrants"/ (70752)
78 "Transients and Migrants"/ (57783)
79 ((alien? or emigrant* or immigrant* or foreigner* or migrant*) adj3 (access* or balanc* or bias* or capacity-building or discrepanc* or discriminat* or disparit* or divers* or equal* or equit* or gap or gaps or harass* or identit* or imbalanc* or inclusi* or inequalit* or in-equal* or inequit* or in-equit* or objectif* or parity or prejudic* or recruit* or represent* or status* or stereotyp* or stereo-typ* or stigma* or underrepresent* or under-represent* or unequal* or un-equal* or vulnerab*)).tw,kw,kf. (14796)
80 Minority Groups/ (37628)
81 Minority Health/ (2089)
82 (minorit* adj3 (access* or balanc* or bias* or capacity-building or discrepanc* or discriminat* or disparit* or divers* or equal* or equit* or gap or gaps or harass* or identit* or imbalanc* or inclusi* or inequalit* or in-equal* or inequit* or in-equit* or objectif* or parity or prejudic* or recruit* or represent* or status* or stereotyp* or stereo-typ* or stigma* or underrepresent* or under-represent* or unequal* or un-equal* or vulnerab*)).tw,kw,kf. (25883)
83 population group?.tw,kw,kf. (34711)
84 (nationality or nationalities).tw,kw,kf. (20353)
85 Race Relations/ (2676)
86 exp Racial Groups/ (574828)
87 ((racial* or race or races or racebased or race-based) adj3 (group* or population? or sector?)).tw,kw,kf. (61750)
88 (racial* or race or races or racebased or race-based).tw,kw,kf. (524985)
89 Racism/ (23139)
90 (antiracial* or anti-racial* or racism* or racist* or antiracis* or anti-racis*).tw,kw,kf. (24757)

91 ((race or races or racial* or biracial* or bi-racial* or interracial* or inter-racial*) adj3 (access* or balanc* or bias* or capacity-building or discrepanc* or discriminat* or disparit* or divers* or equal* or equit* or gap or gaps or harass* or identit* or imbalanc* or inclusi* or inequalit* or in-equal* or inequit* or in-equit* or objectif* or parity or prejudic* or recruit* or represent* or right? or status* or stereotyp* or stereo-typ* or stigma* or underrepresent* or under-represent* or unequal* or un-equal* or vulnerab*)).tw,kw,kf. (116091)
92 Refugees/ (32478)
93 ((refugee? or asylum seeker? or displaced person? or displaced people?) adj3 (access* or balanc* or bias* or capacity-building or discrepanc* or discriminat* or disparit* or divers* or equal* or equit* or gap or gaps or harass* or identit* or imbalanc* or inclusi* or inequalit* or in-equal* or inequit* or in-equit* or objectif* or parity or prejudic* or recruit* or represent* or status* or stereotyp* or stereo-typ* or stigma* or underrepresent* or under-represent* or unequal* or un-equal* or vulnerab*)).tw,kw,kf. (4117)
94 Crime/ (43326)
95 (crime? or criminal* or robber* or steal* or theft* or traffick* or unlawful* or illegal*).tw,kw,kf. (328770)
96 Violence/ (93429)
97 (abus* or assault* or violen*).tw,kw,kf. (573168)
98 ((quality or safe or safety or unsafe) adj3 (accommodat* or communit* or environment* or home? or housing or neighbo?r* or transport* or travel*)).tw,kw,kf. (91925)
99 ((air or atmospher* or environment* or water*) adj3 (clean or condition? or contaminat* or dirty or pollut* or unsanitary)).tw,kw,kf. (516264)
100 Community Participation/ (23741)
101 ((civic or communit* or public or social*) adj3 (action or cohesi* or engag* or exclude? or exclus* or include? or inclus* or involv* or participat* or protect* or support* or vulnerab*)).tw,kw,kf. (437273)
102 adversit*.tw,kw,kf. (33688)
103 or/22-102 [SOCIAL DETERMINANTS OF HEALTH] (7646697)
104 21 and 103 [LONG COVID - SOCIAL DETERMINANTS OF HEALTH] (12724)
105 exp Animals/ not Humans/ (17902502)
106 104 not 105 [ANIMAL-ONLY REMOVED] (12700)
107 (comment or editorial or letter or news or newspaper article).pt. (4678017)
108 106 not 107 [OPINION PIECES REMOVED] (12282)
109 limit 108 to yr="2019-current" (12280)
110 (201912* or 2020* or 2021* or 2022* or 2023* or 2024*).dt. (7472321)
111 109 and 110 [DATE LIMIT APPLIED] (3841)
112 exp Cohort Studies/ (3878750)
113 cohort?.tw,kw,kf. (2624269)
114 (longitudinal* or prospective* or retrospective*).tw,kw,kf. (5941048)
115 (followup or follow-up).tw,kw,kf. (3469151)
116 Observational study.pt. (161897)
117 (observation$2 adj (study or studies)).tw,kw,kf. (485064)
118 ((population or population-based) adj (study or studies or analys#s)).tw,kw,kf. (62245)
119 ((multidimensional or multi-dimensional) adj (study or studies)).tw,kw,kf. (373)
120 Comparative Study.pt. (1928192)
121 ((comparative or comparison) adj (study or studies)).tw,kw,kf. (332518)
122 exp Case-Control Studies/ (1782465)

123 ((case-control* or case-based or case-comparison or case-compeer or case-referrent or case-referent) adj3 (study or studies)).tw,kw,kf. (352658)
124 Cross-Sectional Studies/ (1054865)
125 (crosssection* or cross-section*).tw,kw,kf. (1460273)
126 Multicenter Study/ (760063)
127 ((multicenter or multi-center or multicentre or multi-centre) adj (study or studies)).tw,kw,kf. (162373)
128 or/112-127 [OBSERVATIONAL STUDIES] (13923237)
129 111 and 128 [LONG COVID - SOCIAL DETERMINANTS OF HEALTH - OBSERVATIONAL STUDIES] (1801)
130 (case study or case studies or case report? or case series).ti,kw,kf. (1104392)
131 Case Reports/ (2605796)
132 129 not (130 or 131) [CASE REPORTS REMOVED] (1783)
133 132 use medall [MEDLINE RECORDS] (1783)
134 long COVID/ (12369)
135 post-infectious syndrome/ and coronavirus disease 2019/ (16)
136 ((long* or post*) adj (COVID or COVID-19 or COVID19 or coronavirus* or corona virus*)).tw,kw,kf. (44431)
137 ((long-term or longterm or post-acute or postacute or chronic) adj3 (COVID or COVID-19 or COVID19 or coronavirus* or corona virus* or 2019-nCoV or 19nCoV or 2019nCoV or SARS-CoV-2 or SARS-CoV2 or SARSCoV-2 or SARSCoV2 or SARS2 or SARS-2 or severe acute respiratory syndrome coronavirus 2)).tw,kw,kf. (12987)
138 (("long* haul*" or longhaul* or "long* tail*" or longtail* or longduration* or "long duration*" or longlast* or "long last*" or longstanding* or "long standing*" or "medium* term*" or mediumterm*) adj3 (COVID or COVID-19 or COVID19 or coronavirus* or corona virus* or 2019-nCoV or 19nCoV or 2019nCoV or SARS-CoV-2 or SARS-CoV2 or SARSCoV-2 or SARSCoV2 or SARS2 or SARS-2 or severe acute respiratory syndrome coronavirus 2)).tw,kw,kf. (1169)
139 ((nonrecover* or "non recover*" or "not recover*") adj3 (COVID or COVID-19 or COVID19 or coronavirus* or corona virus* or 2019-nCoV or 19nCoV or 2019nCoV or SARS-CoV-2 or SARS-CoV2 or SARSCoV-2 or SARSCoV2 or SARS2 or SARS-2 or severe acute respiratory syndrome coronavirus 2)).tw,kw,kf. (47)
140 ((after discharg* or following discharg* or postacute* or "post acute*" or postdischarg* or "post discharge" or "post discharging" or posthospital* or post-hospital* or postinfect* or "post infection" or "post infective*" or postviral* or "post viral*" or postvirus* or "post virus*" or postcritical or post-critical or postintensive or post-intensive or post-ICU) adj3 (complication? or consequence? or convalescen* or disabilit* or feature* or illness* or prognos* or sequela* or sign or signs or suffering? or symptom* or recuperat*) adj3 (COVID or COVID-19 or COVID19 or coronavirus* or corona virus* or 2019-nCoV or 19nCoV or 2019nCoV or SARS-CoV-2 or SARS-CoV2 or SARSCoV-2 or SARSCoV2 or SARS2 or SARS-2 or severe acute respiratory syndrome coronavirus 2)).tw,kw,kf. (3252)
141 ((chronic* or continuous* or continual* or continuing* or delay* or endur* or extend* or fluctuat* or gradual* or lasting* or legacy* or lengthy* or linger* or long* or "medium* term*" or mediumterm* or multisystem* or "multi system*" or ongoing or permanent* or persist* or prolong* or protract* or relaps* or remission* or remit* or residual* or slow* or subacute* or "sub acute*") adj3 (complication? or consequence? or convalescen* or disabilit* or feature* or illness* or prognos* or sequela* or sign or signs or suffering? or symptom* or recuperat*) adj3 (COVID or COVID-19 or COVID19 or coronavirus* or corona virus* or 2019-nCoV or 19nCoV or 2019nCoV or

SARS-CoV-2 or SARS-CoV2 or SARSCoV-2 or SARSCoV2 or SARS2 or SARS-2 or severe acute respiratory syndrome coronavirus 2)).tw,kw,kf. (8713)
142 ((after discharg* or following discharg* or postacute* or "post acute*" or postdischarg* or "post discharge" or "post discharging" or posthospital* or post-hospital* or postinfect* or "post infection" or "post infective*" or postviral* or "post viral*" or postvirus* or "post virus*" or postcritical or post-critical or postintensive or post-intensive or post-ICU) adj3 recover* adj3 (COVID or COVID-19 or COVID19 or coronavirus* or corona virus* or 2019-nCoV or 19nCoV or 2019nCoV or SARS-CoV-2 or SARS-CoV2 or SARSCoV-2 or SARSCoV2 or SARS2 or SARS-2 or severe acute respiratory syndrome coronavirus 2)).tw,kw,kf. (104)
143 ((chronic* or continuous* or continual* or continuing* or delay* or endur* or extend* or fluctuat* or gradual* or lasting* or legacy* or lengthy* or linger* or long* or "medium* term*" or mediumterm* or multisystem* or "multi system*" or ongoing* or permanent* or persist* or prolong* or protract* or relaps* or remission* or remit* or residual* or slow* or subacute* or "sub acute*") adj3 recover* adj3 (COVID or COVID-19 or COVID19 or coronavirus* or corona virus* or 2019-nCoV or 19nCoV or 2019nCoV or SARS-CoV-2 or SARS-CoV2 or SARSCoV-2 or SARSCoV2 or SARS2 or SARS-2 or severe acute respiratory syndrome coronavirus 2)).tw,kw,kf. (706)
144 (following adj (COVID or COVID-19 or COVID19 or coronavirus* or corona virus* or 2019-nCoV or 19nCoV or 2019nCoV or SARS-CoV-2 or SARS-CoV2 or SARSCoV-2 or SARSCoV2 or SARS2 or SARS-2 or severe acute respiratory syndrome coronavirus 2) adj3 (comorbid* or "co morbid*" or condition* or convalescen* or disease* or disorder* or illness* or multimorbid* or "multi morbid*" or sickness* or symptom* or syndrome* or sign or signs or prognos* or recuperat* or survivor* or survival* or risk*)).tw,kw,kf. (2471)
145 (after adj (COVID or COVID-19 or COVID19 or coronavirus* or corona virus* or 2019-nCoV or 19nCoV or 2019nCoV or SARS-CoV-2 or SARS-CoV2 or SARSCoV-2 or SARSCoV2 or SARS2 or SARS-2 or severe acute respiratory syndrome coronavirus 2) adj3 (comorbid* or "co morbid*" or condition* or convalescen* or disease* or disorder* or illness* or multimorbid* or "multi morbid*" or sickness* or symptom* or syndrome* or sign or signs or prognos* or recuperat* or survivor* or survival* or risk*)).tw,kw,kf. (4418)
146 or/134-145 [LONG COVID PT 1] (59110)
147 COVID-19/ (438515)
148 (COVID or COVID-19 or COVID19 or coronavirus* or corona virus* or 2019-nCoV or 19nCoV or 2019nCoV or SARS-CoV-2 or SARS-CoV2 or SARSCoV-2 or SARSCoV2 or SARS2 or SARS-2 or severe acute respiratory syndrome coronavirus 2).ti,kw,kf. (837927)
149 or/147-148 (905002)
150 convalescence/ (69581)
151 149 and 150 [LONG COVID PT 2] (3925)
152 146 or 151 [LONG COVID PTS 1-2] (62340)
153 "social determinants of health"/ (33577)
154 ((social* or socioeconomic* or socio-economic*) adj determin*).tw,kw,kf. (51239)
155 ((commercial* or structural*) adj3 determin* adj3 health*).tw,kw,kf. (2003)
156 ((nonmedical or non-medical) adj (factor? or variables)).tw,kw,kf. (1170)
157 "social aspects and related phenomena"/ (1156)
158 social aspect/ (89122)
159 ((social or sociologic*) adj3 (arrangement? or class$2 or condition? or environment? or gradient? or situation?)).tw,kw,kf. (101440)
160 ((social or sociologic*) adj3 (aspect? or caus* or component? or consider* or detail? or effect? or element* or facet? or factor? or feature? or influen* or patient or patients or reason* or variables)).tw,kw,kf. (254356)

161 social problem/ (30589)
162 exp social status/ (269847)
163 exp housing quality/ (1895)
164 housing instability/ (1002)
165 home security/ (16)
166 ((accommodat* or housing or home? or living) adj3 (arrangement? or condition? or environment? or factor? or situation? or variables)).tw,kw,kf. (113799)
167 ((accommodat* or housing or home? or living) adj3 (access* or availabl* or insecur* or secur* or instabilit* or stabilit* or stable or unstable or precarious* or marginal* or safe or safety or unsafe or vulnerab*)).tw,kw,kf. (38541)
168 exp food insecurity/ (12631)
169 food security/ (11494)
170 ((food or grocer* or nutritio*) adj3 (access* or availab* or insecur* or secur* or instabilit* or stabilit* or stable or unstable or suppli* or supply* or precarious* or marginal* or safe or safety or unsafe or vulnerab*)).tw,kw,kf. (172483)
171 ((exercis* or physical activit* or sport*) adj3 (access* or availab* or insecur* or secur* or instabilit* or stabilit* or stable or unstable or suppli* or supply* or opportunit* or precarious* or marginal* or safe or safety or unsafe)).tw,kw,kf. (23159)
172 (amenity or amenities).tw,kw,kf. (4255)
173 employment/ (140010)
174 employment status/ (76851)
175 unemployment/ (38181)
176 job security/ (1333)
177 ((employ* or job or jobs or work*) adj3 (abilit* or access* or availab* or insecur* or secur* or instabilit* or stabilit* or stable or unstable or condition? or opportunit* or precarious* or marginal* or safe or safety or unsafe or vulnerab*)).tw,kw,kf. (205916)
178 unemploy*.tw,kw,kf. (58842)
179 sociodemographics/ (26715)
180 ((sociodemograph* or socio-demograph*) adj3 (aspect? or caus* or component? or consider* or detail? or effect? or element* or facet? or factor? or feature? or influen* or patient or patients or reason* or variables)).tw,kw,kf. (111759)
181 socioeconomics/ (177895)
182 exp socioeconomic vulnerability/ (1610)
183 ((socioeconomic* or socio-economic*) adj3 (aspect? or caus* or component? or consider* or detail? or effect? or element* or facet? or factor? or feature? or influen* or patient or patients or reason* or variables)).tw,kw,kf. (107229)
184 economic status/ (4000)
185 ((economic* or financ* or income*) adj3 (insecur* or secur* or instabilit* or stabilit* or stable or unstable or vulnerab*)).tw,kw,kf. (22863)
186 (economic* adj3 (condition? or environment? or factor? or situation? or variables)).tw,kw,kf. (78734)
187 exp poverty/ (111915)
188 (poverty or impoverish* or limited income? or low* income? or reduced income?).tw,kw,kf. (222047)
189 exp academic achievement/ (49954)
190 educational status/ (185353)

191 ((academic* or education* or highschool* or high-school* or school*) adj3 (abilit* or achiev* or attain* or background* or enroll* or failure? or graduat* or level? or quality or status or success*)).tw,kw,kf. (434887)
192 illiteracy/ (3228)
193 literacy/ (9563)
194 (literate or literac* or illiterate* or illiterac*).tw,kw,kf. (97877)
195 ((read* or writ*) adj3 (abilit* or limit* or proficienc* or skill*)).tw,kw,kf. (28938)
196 ((language? or speak*) adj3 (abilit* or limit* or proficienc* or skill*)).tw,kw,kf. (39762)
197 exp English proficiency/ (1656)
198 digital divide/ (1136)
199 (digital adj (divide or division?)).tw,kw,kf. (2862)
200 ((earl* child* or earl* year?) adj3 (develop* or educat*)).tw,kw,kf. (12151)
201 exp health care access/ (130192)
202 ((care or healthcare or health care or health service?) adj3 (access* or availab* or equit*)).tw,kw,kf. (215655)
203 exp health literacy/ (33996)
204 ((health or healthcare) adj3 (literac* or literate)).tw,kw,kf. (42035)
205 colorism/ (80)
206 prejudice/ (30417)
207 social discrimination/ (8046)
208 disability discrimination/ (390)
209 employment discrimination/ (598)
210 insurance discrimination/ (35)
211 exp "discrimination against sexual and gender minorities"/ (1945)
212 (discriminat* or prejudic*).tw,kw,kf. (732323)
213 (antisemit* or anti-semit* or islamophobi* or islamo-phobi*).tw,kw,kf. (919)
214 xenophobia/ (548)
215 (xenophobi* or xeno-phobi*).tw,kw,kf. (1049)
216 exp incarceration/ (14966)
217 exp prisoner/ (40579)
218 (incarcerat* or prison* or imprison* or jail*).tw,kw,kf. (85614)
219 exp ethnic group/ (318522)
220 exp "ethnic or racial aspects"/ (372989)
221 ((ethnic* or nationalit*) adj3 (access* or balanc* or bias* or capacity-building or discrepanc* or discriminat* or disparit* or divers* or equal* or equit* or gap or gaps or harass* or identit* or imbalanc* or inclusi* or inequalit* or in-equal* or inequit* or in-equit* or objectif* or parity or prejudic* or recruit* or represent* or status* or stereotyp* or stereo-typ* or stigma* or underrepresent* or under-represent* or unequal* or un-equal* or vulnerab*)).tw,kw,kf. (96964)
222 ethnic*.tw,kw,kf. (492505)
223 exp migrant/ (69330)
224 ((alien? or emigrant* or immigrant* or foreigner* or migrant*) adj3 (access* or balanc* or bias* or capacity-building or discrepanc* or discriminat* or disparit* or divers* or equal* or equit* or gap or gaps or harass* or identit* or imbalanc* or inclusi* or inequalit* or in-equal* or inequit* or in-equit* or objectif* or parity or prejudic* or recruit* or represent* or status* or stereotyp* or stereo-typ* or stigma* or underrepresent* or under-represent* or unequal* or un-equal* or vulnerab*)).tw,kw,kf. (14796)
225 exp minority group/ (86119)
226 minority health/ (2089)

227 (minorit* adj3 (access* or balanc* or bias* or capacity-building or discrepanc* or discriminat* or disparit* or divers* or equal* or equit* or gap or gaps or harass* or identit* or imbalanc* or inclusi* or inequalit* or in-equal* or inequit* or in-equit* or objectif* or parity or prejudic* or recruit* or represent* or status* or stereotyp* or stereo-typ* or stigma* or underrepresent* or under-represent* or unequal* or un-equal* or vulnerab*)).tw,kw,kf. (25883)
228 population group?.tw,kw,kf. (34711)
229 (nationality or nationalities).tw,kw,kf. (20353)
230 exp race relation/ (28647)
231 ((racial* or race or races or racebased or race-based) adj3 (group* or population? or sector?)).tw,kw,kf. (61750)
232 (racial* or race or races or racebased or race-based).tw,kw,kf. (524985)
233 exp racism/ (24918)
234 (antiracial* or anti-racial* or racism* or racist* or antiracis* or anti-racis*).tw,kw,kf. (24757)
235 ((race or races or racial* or biracial* or bi-racial* or interracial* or inter-racial*) adj3 (access* or balanc* or bias* or capacity-building or discrepanc* or discriminat* or disparit* or divers* or equal* or equit* or gap or gaps or harass* or identit* or imbalanc* or inclusi* or inequalit* or in-equal* or inequit* or in-equit* or objectif* or parity or prejudic* or recruit* or represent* or right? or status* or stereotyp* or stereo-typ* or stigma* or underrepresent* or under-represent* or unequal* or un-equal* or vulnerab*)).tw,kw,kf. (116091)
236 refugee/ (32478)
237 ((refugee? or asylum seeker? or displaced person? or displaced people?) adj3 (access* or balanc* or bias* or capacity-building or discrepanc* or discriminat* or disparit* or divers* or equal* or equit* or gap or gaps or harass* or identit* or imbalanc* or inclusi* or inequalit* or in-equal* or inequit* or in-equit* or objectif* or parity or prejudic* or recruit* or represent* or status* or stereotyp* or stereo-typ* or stigma* or underrepresent* or under-represent* or unequal* or un-equal* or vulnerab*)).tw,kw,kf. (4117)
238 exp crime/ (277441)
239 (crime? or criminal* or robber* or steal* or theft* or traffick* or unlawful* or illegal*).tw,kw,kf. (328770)
240 exp abuse/ (268655)
241 exp violence/ (309079)
242 (abus* or assault* or violen*).tw,kw,kf. (573168)
243 ((quality or safe or safety or unsafe) adj3 (accommodat* or communit* or environment* or home? or housing or neighbo?r* or transport* or travel*)).tw,kw,kf. (91925)
244 water insecurity/ (1173)
245 ((air or atmospher* or environment* or water*) adj3 (clean or condition? or contaminat* or dirty or pollut* or unsanitary)).tw,kw,kf. (516264)
246 community participation/ (23741)
247 ((civic or communit* or public or social*) adj3 (action or cohesi* or engag* or exclude? or exclus* or include? or inclus* or involv* or participat* or protect* or support* or vulnerab*)).tw,kw,kf. (437273)
248 exp childhood adversity/ (69904)
249 adversit*.tw,kw,kf. (33688)
250 or/153-249 [SOCIAL DETERMINANTS OF HEALTH] (6171558)
251 152 and 250 [LONG COVID - SOCIAL DETERMINANTS OF HEALTH] (9845)
252 (exp animal/ or exp animal model/ or exp animal experiment/ or nonhuman/ or exp vertebrate/) not (exp human/ or exp human experiment/) (13604010)
253 251 not 252 [ANIMAL-ONLY REMOVED] (9815)

254 (editorial or letter).pt. (4147267)
255 253 not 254 [OPINION PIECES REMOVED] (9527)
256 limit 255 to yr="2019-current" (9525)
257 (201912* or 2020* or 2021* or 2022* or 2023* or 2024*).dc. (9701143)
258 256 and 257 [DATE LIMIT APPLIED] (5822)
259 cohort analysis/ (1571538)
260 cohort?.tw,kw,kf. (2624269)
261 longitudinal study/ (398954)
262 prospective study/ (1643876)
263 retrospective study/ (2926172)
264 (longitudinal* or prospective* or retrospective*).tw,kw,kf. (5941048)
265 follow up/ (2294512)
266 (followup or follow-up).tw,kw,kf. (3469151)
267 observational study/ (556617)
268 (observation$2 adj (study or studies)).tw,kw,kf. (485064)
269 population research/ (143143)
270 ((population or population-based) adj (study or studies or analys#s)).tw,kw,kf. (62245)
271 ((multidimensional or multi-dimensional) adj (study or studies)).tw,kw,kf. (373)
272 exp comparative study/ (3729054)
273 ((comparative or comparison) adj (study or studies)).tw,kw,kf. (332518)
274 exp case control study/ (1782465)
275 ((case-control* or case-based or case-comparison or case-compeer or case-referrent or case-referent) adj3 (study or studies)).tw,kw,kf. (352658)
276 cross-sectional study/ (1183847)
277 (crosssection* or cross-section*).tw,kw,kf. (1460273)
278 multicenter study/ (760063)
279 ((multicenter or multi-center or multicentre or multi-centre) adj (study or studies)).tw,kw,kf. (162373)
280 or/259-279 [OBSERVATIONAL STUDIES] (15808087)
281 258 and 280 [LONG COVID - SOCIAL DETERMINANTS OF HEALTH - OBSERVATIONAL STUDIES] (3337)
282 (case study or case studies or case report? or case series).ti,kw,kf. (1104392)
283 case report/ (3148039)
284 exp case study/ (2549199)
285 281 not (282 or 283 or 284) [CASE REPORTS REMOVED] (3230)
286 conference abstract.pt. (5245291)
287 285 not 286 [CONFERENCE ABSTRACTS REMOVED] (2596)
288 287 use emczd [EMBASE RECORDS] (2596)
289 133 or 288 [BOTH DATABASES] (4379)
290 remove duplicates from 289 (2947) [**TOTAL UNIQUE RECORDS**]
291 290 use medall [MEDLINE UNIQUE RECORDS] (1767)
292 290 use emczd [EMBASE UNIQUE RECORDS] (1180)

****
Web of Science (*Core Collection excluding Conference Databases*)

Set #	Search Query	Results

1	(long* or post*) NEAR/0 (COVID or "COVID-19" or COVID19 or coronavirus* or "corona virus" or "corona viruses") (Topic) OR ("long-term" or longterm or "post-acute" or postacute or chronic) NEAR/3 (COVID or "COVID-19" or COVID19 or coronavirus* or "corona virus" or "corona viruses" or "2019-nCoV" or 19nCoV or 2019nCoV or "SARS-CoV-2" or "SARS-CoV2" or "SARSCoV-2" or SARSCoV2 or SARS2 or "SARS-2" or "severe acute respiratory syndrome coronavirus 2") (Topic) OR ("long haul" or "longer haul" or "long hauler" or "long haulers" or longhaul* or "long tail" or "longer tail" or "long tails" or longtail* or longduration* or "long duration" or "long durations" or longlast* or "long lasting" or longstanding* or "long standing" or "medium term" or "medium terms" or mediumterm*) NEAR/3 (COVID or "COVID-19" or COVID19 or coronavirus* or "corona virus" or "corona viruses" or "2019-nCoV" or 19nCoV or 2019nCoV or "SARS-CoV-2" or "SARS-CoV2" or "SARSCoV-2" or SARSCoV2 or SARS2 or "SARS-2" or "severe acute respiratory syndrome coronavirus 2") (Topic) OR (nonrecover* or "non recover" or "non recovery" or "non recoveries" or "not recover" or "not recovered") NEAR/3 (COVID or "COVID-19" or COVID19 or coronavirus* or "corona virus" or "corona viruses" or "2019-nCoV" or 19nCoV or 2019nCoV or "SARS-CoV-2" or "SARS-CoV2" or "SARSCoV-2" or SARSCoV2 or SARS2 or "SARS-2" or "severe acute respiratory syndrome coronavirus 2"). (Topic) Editions: WOS.SCI,WOS.SSCI,WOS.AHCI,WOS.BSCI,WOS.BHCI,WOS.ESCI	29202
2	(social* or socioeconomic* or "socio-economic" or "socio-economics" or "socio-economical" or "socio-economically") NEAR/0 determin* (Topic) OR (commercial* or structural*) NEAR/3 determin* (Topic) OR (nonmedical or "non-medical") NEAR/0 (factor or factors or variable*) (Topic) OR (social or sociologic*) NEAR/3 (arrangement* or class or classes or condition or conditions or environment* or gradient* or situation*) (Topic) OR (social or sociologic*) NEAR/3 (aspect or aspects or caus* or component* or consider* or detail or details or effect or effects or element* or facet or facets or factor or factors or feature or features or influen* or patient or patients or reason* or variable*) (Topic) Editions: WOS.SCI,WOS.SSCI,WOS.AHCI,WOS.BSCI,WOS.BHCI,WOS.ESCI	493354

3	(accommodat* or housing or home or homes or living) NEAR/3 (arrangement* or condition or conditions or environment* or factor or factors or situation* or variable*) (Topic) OR (accommodat* or housing or home or homes or living) NEAR/3 (access* or availabl* or insecur* or secur* or instabilit* or stabilit* or stable or unstable or precarious* or marginal* or safe or safety or unsafe or vulnerab*) (Topic) OR (food or grocer* or nutritio*) NEAR/3 (access* or availab* or insecur* or secur* or instabilit* or stabilit* or stable or unstable or suppli* or supply* or precarious* or marginal* or safe or safety or unsafe or vulnerab*) (Topic) OR (exercis* or "physical activity" or "physical activities" or sport*) NEAR/3 (access* or availab* or insecur* or secur* or instabilit* or stabilit* or stable or unstable or suppli* or supply* or opportunit* or precarious* or marginal* or safe or safety or unsafe) (Topic) OR amenity or amenities (Topic) Editions: WOS.SCI,WOS.SSCI,WOS.AHCI,WOS.BSCI,WOS.BHCI,WOS.ESCI	394215
4	(employ* or job or jobs or work*) NEAR/3 (abilit* or access* or availab* or insecur* or secur* or instabilit* or stabilit* or stable or unstable or condition or conditions or opportunit* or precarious* or marginal* or safe or safety or unsafe or vulnerab*) (Topic) OR unemploy* (Topic) OR (sociodemograph* or "socio-demographic" or "socio-demographics" or "socio-demographical" or "socio-demography") NEAR/3 (aspect or aspects or caus* or component* or consider* or detail or details or effect or effects or element* or facet or facets or factor or factors or feature or features or influen* or patient or patients or reason* or variable*) (Topic) OR (socioeconomic* or "socio-economic" or "socio-economical" or "socio-economically" or "socio-economics") NEAR/3 (aspect or aspects or caus* or component* or consider* or detail or details or effect or effects or element* or facet or facets or factor or factors or feature or features or influen* or patient or patients or reason* or variable*) (Topic) OR (economic* or financ* or income*) NEAR/3 (insecur* or secur* or instabilit* or stabilit* or stable or unstable or vulnerab*). (Topic) OR poverty or impoverish* or "limited income" or "limited incomes" or "low income" or "low incomes" or "lower income" or "lower incomes" or "lowest income" or "lowest incomes" or "reduced income"or "reduced incomes" (Topic) Editions: WOS.SCI,WOS.SSCI,WOS.AHCI,WOS.BSCI,WOS.BHCI,WOS.ESCI	741687

5	(academic* or education* or highschool* or high-school* or school*) NEAR/3 (abilit* or achiev* or attain* or background* or enroll* or failure or failures or graduat* or level or levels or quality or status or success*) (Topic) OR literate or literac* or illiterate* or illiterac* (Topic) OR (read* or writ*) NEAR/3 (abilit* or limit* or proficienc* or skill*) (Topic) OR (language* or speak*) NEAR/3 (abilit* or limit* or proficienc* or skill*) (Topic) OR digital NEAR/0 (divide or division or divisions) (Topic) OR ("early child" or "early children" or "earlier child" or "earlier children" or "earliest child" or "earliest children" or "early year" or "early years" or "earlier year" or "earlier years" or "earliest year" or "earliest years") NEAR/3 (develop* or educat*) (Topic) Editions: WOS.SCI,WOS.SSCI,WOS.AHCI,WOS.BSCI,WOS.BHCI,WOS.ESCI	561204
6	(care or healthcare or "health care" or "health service" or "health services") NEAR/3 (access* or availab* or equit*) (Topic) OR discriminat* or prejudic* (Topic) OR antisemit* or anti-semit* or islamophobi* or islamo-phobi* (Topic) OR xenophobi* or xeno-phobi* (Topic) OR incarcerat* or prison* or imprison* or jail* (Topic) Editions: WOS.SCI,WOS.SSCI,WOS.AHCI,WOS.BSCI,WOS.BHCI,WOS.ESCI	815287
7	(ethnic* or nationalit*) NEAR/3 (access* or balanc* or bias* or "capacity-building" or discrepanc* or discriminat* or disparit* or divers* or equal* or equit* or gap or gaps or harass* or identit* or imbalanc* or inclusi* or inequalit* or in-equal* or inequit* or in-equit* or objectif* or parity or prejudic* or recruit* or represent* or status* or stereotyp* or stereo-typ* or stigma* or underrepresent* or under-represent* or unequal* or un-equal* or vulnerab*) (Topic) OR ethnic* (Topic) OR (alien or aliens or emigrant* or immigrant* or foreigner* or migrant*) NEAR/3 (access* or balanc* or bias* or capacity-building or discrepanc* or discriminat* or disparit* or divers* or equal* or equit* or gap or gaps or harass* or identit* or imbalanc* or inclusi* or inequalit* or in-equal* or inequit* or in-equit* or objectif* or parity or prejudic* or recruit* or represent* or status* or stereotyp* or stereo-typ* or stigma* or underrepresent* or under-represent* or unequal* or un-equal* or vulnerab*) (Topic) OR minorit* NEAR/3 (access* or balanc* or bias* or capacity-building or discrepanc* or discriminat* or disparit* or divers* or equal* or equit* or gap or gaps or harass* or identit* or imbalanc* or inclusi* or inequalit* or in-equal* or inequit* or in-equit* or objectif* or parity or prejudic* or recruit* or represent* or status* or stereotyp* or stereo-typ* or stigma* or underrepresent* or under-represent* or unequal* or un-equal* or vulnerab*) (Topic) OR nationality or nationalities (Topic) OR "population group" or "population groups" (Topic) Editions: WOS.SCI,WOS.SSCI,WOS.AHCI,WOS.BSCI,WOS.BHCI,WOS.ESCI	408128

8	(racial* or race or races or racebased or "race-based") NEAR/3 (group* or population? or sector or sectors) (Topic) OR racial* or race or races or racebased or race-based (Title) OR antiracial* or anti-racial* or racism* or racist* or antiracis* or anti-racis* (Topic) OR (race or races or racial* or biracial* or bi-racial* or interracial* or inter-racial*) NEAR/3 (access* or balanc* or bias* or capacity-building or discrepanc* or discriminat* or disparit* or divers* or equal* or equit* or gap or gaps or harass* or identit* or imbalanc* or inclusi* or inequalit* or in-equal* or inequit* or in-equit* or objectif* or parity or prejudic* or recruit* or represent* or right or rights or status* or stereotyp* or stereo-typ* or stigma* or underrepresent* or under-represent* or unequal* or un-equal* or vulnerab*) (Topic) OR (refugee* or "asylum seeker" or "asylum seekers" or "displaced person" or "displaced persons" or "displaced people" or "displaced peoples") NEAR/3 (access* or balanc* or bias* or "capacity-building" or discrepanc* or discriminat* or disparit* or divers* or equal* or equit* or gap or gaps or harass* or identit* or imbalanc* or inclusi* or inequalit* or in-equal* or inequit* or in-equit* or objectif* or parity or prejudic* or recruit* or represent* or status* or stereotyp* or stereo-typ* or stigma* or underrepresent* or under-represent* or unequal* or un-equal* or vulnerab*) (Topic) Editions: WOS.SCI,WOS.SSCI,WOS.AHCI,WOS.BSCI,WOS.BHCI,WOS.ESCI	269099
9	crime or crimes or criminal* or robber* or steal* or theft* or traffick* or unlawful* or illegal* (Topic) OR abus* or assault* or violen* (Topic) OR (quality or safe or safety or unsafe) NEAR/3 (accommodat* or communit* or environment* or home or homes or housing or neighbor* or neighbour* or transport* or travel*) (Topic) OR (air or atmospher* or environment* or water*) NEAR/3 (clean or condition or conditions or contaminat* or dirty or pollut* or unsanitary) (Topic) OR (civic or communit* or public or social*) NEAR/3 (action or cohesi* or engag* or exclude or excluded or excludes or exclus* or include or included or includes or inclus* or involv* or participat* or protect* or support* or vulnerab*) (Topic) OR adversit* (Topic) Editions: WOS.SCI,WOS.SSCI,WOS.AHCI,WOS.BSCI,WOS.BHCI,WOS.ESCI	2179288
10	#2 OR #3 OR #4 OR #5 OR #6 OR #7 OR #8 OR #9 Editions: WOS.SCI,WOS.SSCI,WOS.AHCI,WOS.BSCI,WOS.BHCI,WOS.ESCI	4965858
11	#10 AND #1 Editions: WOS.SCI,WOS.SSCI,WOS.AHCI,WOS.BSCI,WOS.BHCI,WOS.ESCI	5014
12	cohort or cohorts (Topic) OR longitudinal* or prospective* or retrospective* (Topic) OR followup or "follow-up" (Topic) Editions: WOS.SCI,WOS.SSCI,WOS.AHCI,WOS.BSCI,WOS.BHCI,WOS.ESCI	4102214

13	observation* NEAR/0 (study or studies) (Topic) OR (population or "population-based") NEAR/0 (study or studies or analyses or analysis) (Topic) OR (multidimensional or "multi-dimensional") NEAR/0 (study or studies or analyses or analysis) (Topic) OR (comparative or comparison) NEAR/0 (study or studies) (Topic) Editions: WOS.SCI,WOS.SSCI,WOS.AHCI,WOS.BSCI,WOS.BHCI,WOS.ESCI	685295
14	(case-control* or "case-based" or "case-comparison" or "case-compeer" or "case-referrent" or "case-referent") NEAR/3 (study or studies) (Topic) OR crosssection* or (cross NEAR/0 section*) (Topic) OR (multidimensional or "multi-dimensional") NEAR/0 (study or studies or analyses or analysis) (Topic) OR (multicenter or "multi-center" or multicentre or "multi-centre") NEAR/0 (study or studies) (Topic) Editions: WOS.SCI,WOS.SSCI,WOS.AHCI,WOS.BSCI,WOS.BHCI,WOS.ESCI	1212380
15	#14 OR #13 OR #12 Editions: WOS.SCI,WOS.SSCI,WOS.AHCI,WOS.BSCI,WOS.BHCI,WOS.ESCI	5478327
16	#11 AND #15 Editions: WOS.SCI,WOS.SSCI,WOS.AHCI,WOS.BSCI,WOS.BHCI,WOS.ESCI	1512
17	"case study" or "case studies" or "case report" or "case reports" (Title) Editions: WOS.SCI,WOS.SSCI,WOS.AHCI,WOS.BSCI,WOS.BHCI,WOS.ESCI	611631
18	#16 NOT #17 Editions: WOS.SCI,WOS.SSCI,WOS.AHCI,WOS.BSCI,WOS.BHCI,WOS.ESCI	1505
