# Supplementary material for: Understanding how social determinants of health shape Long COVID outcomes: a rapid review of evidence
Source: Arch Public Health. 2025 Dec 29;83:308. doi: 10.1186/s13690-025-01787-x (PMC12751510; doi:10.1186/s13690-025-01787-x)
Supplement: Supplementary file 3 — Additional file 3. Summary of study design, population, geographic location, definitions of Long COVID, social determinants examined, and key findings extracted from each included study. [file 13690_2025_1787_MOESM3_ESM.pdf]

Additional File 3. Summary of study design, population, geographic location, definitions of Long COVID, social determinants examined, and key findings extracted from each included study

Number	Author's Last name	Country	Study design	Population type	Sample Size	Main Findings
1	Adler et al., 2023	Israel	Cross-sectional	Children (5–18 yrs) with/without prior COVID-19	3240 individuals	Older children (12–18 years) had higher odds of reporting Long COVID symptoms each year of age associated with an 8% increased risk (OR=1.08; 95% CI: 1.03–1.14; p=0.001).
2	Atchison et al., 2023	United Kingdom	Cross-sectional	Children (5–17 yrs) with symptomatic prior COVID-19 >3 months prior	10,059 individuals	Older children (12–17 years) had higher odds of reporting persistent symptoms (OR= 3.4 95% CI: 2.8 - 4.0)
3	Azambuja et al., 2024	Brazil	Cohort	Socially vulnerable adults (18 +yrs) with COVID-19 >3 months prior	710 participants	Females had a higher likelihood of reporting incomplete recovery (OR= 2.15, CI: 1.31–3.64, p = 0.0032), worsening dyspnoea (OR= 2.14, CI: 1.26–3.76, p = 0.0060), poorer health-related quality of life (OR= 2.46, CI: 1.60–3.83, p = 0.0001), and functional impairment (OR= 1.57, CI: 1.01–2.46, p = 0.046). Primary education or lower had an increased risk of reduced quality of life (OR: 1.80, CI: 1.17–2.76, p = 0.0070)
4	Bonner et al., 2024	United States	Cross-sectional	Adults (18–64 yrs, U.S. workforce) with COVID-19 >3 months prior	18,816 individuals	Females were 1.43 times more likely to develop Long COVID (95% CI: 1.22–1.67, p < 0.001).
5	Bovil et al., 2022	27 European countries and Israel	Cross-sectional	Adults (50 +yrs) with confirmed COVID-19	3,156 individuals	Medium education (OR=2.4 CI 1.39–4.09, p <0.01), low education (OR= 2.14 95% CI 1.18–3.86, p < 0.05), and ages 70 +yrs (OR= 1.61, 95% CI 1.08–

						2.41, p <0.05) have a higher risk of post-COVID-19 conditions
6	Chelly et al., 2023	Tunisia	Cross-sectional	Adults (18 +yrs) with confirmed COVID-19	1,381 individuals	Female sex was (OR = 1.66, 95% CI 1.21–2.28, p = 0.02), and age ≥ 60 years (OR = 2.34, 95% CI 1.00–5.49, p = 0.049) were predictive factor of long COVID.
7	Chilunga et al., 2023	Netherlands	Cohort	Hospitalized patients (18+ yrs) with confirmed COVID-19	1,886 individuals	Females (PR = 1.26, 95% CI 1.08–1.48)., African Surinamese (PR = 1.41, 95% CI 1.10–1.82), South Asian Surinamese (PR = 1.54, 95% CI 1.07–2.21), Moroccan (PR = 1.39, 95% CI 1.05–1.83), and Turkish origin (PR = 1.45, 95% CI 1.04–2.02) were predictors of long COVID
8	Chudzik et al., 2022	Poland	Cohort	Adults (18+ yrs) with post-COVID symptoms seeking care	2,218 individuals	Females (OR = 1.48, 95% CI: 1.19–1.84, p < 0.001) is a risk factor for long COVID
9	Coste et al., 2024	France	Cross-sectional	Adults (18+ yrs) with/without COVID-19 history	10,615 individuals	Women (PR=2.18, 95% CI: 1.68–2.83) and unemployed individuals experienced greater prevalence of long COVID (PR= 1.68 95%CI 1.08-2.64)
10	Crankson et al., 2022	Ghana	Cross-sectional	COVID-19 patients (30–59 yrs)	2,334 individuals	Women are less likely to be diagnosed with long COVID than men (OR = 0.52, 95% CI 0.27–0.99, p = 0.05). primary (OR = 0.73, 95% CI = 0.01–0.66, p = 0.02), secondary/vocational (OR = 0.26, 95% CI = 0.09–0.77, p = 0.02) and tertiary education (OR = 0.23, 95% CI = 0.07–0.72, p = 0.12) had lower odds of

						long COVID diagnosis compared to those with no formal education
11	Daniel et al., 2023	United States	Cohort	Adults (18 +yrs) with confirmed COVID-19 diagnosis	516 individuals	Females with commercial insurance had significantly lower odds of long COVID (OR = 0.18, 95% CI: 0.03–0.96, p= 0.0173)
12	Durstenfeld et al., 2023	United States	Cohort	Adults (18+ yrs) COVID-19 positive in Citizen Science study	13,305 individuals	Lower socioeconomic status/financial insecurity (OR, 1.62; 95% CI, 1.02–2.63, p=0.04) were associated with Long COVID symptoms
13	Eligulashvili et al., 2024	United States	Cohort	Adults (18+ yrs) with prolonged COVID-19 symptoms	52,732 individuals (643 CORE with prolonged symptoms, 52,089 non-CORE for comparison)	Unmet health-related social needs were significantly linked to worse long COVID symptoms. Among CORE patients, 29.81% had at least one unmet HRSN. These patients reported higher rates of brain fog (42.73% vs. 32.05%, p = 0.049), more severe tiredness (median ESAS-r score 7 vs. 5, p = 0.002), and greater pain (5 vs. 3, p < 0.001). Patients from the lowest-income quintile (≤ $55,275.80 median household income) were more prevalent in the Long COVID group (53.81% vs. 43.67%, p < 0.001).
14	Feldstein et al., 2023	United States	Cross-sectional	Adults (18+ yrs) with self-reported COVID-19 test status	2,890 individuals	Hispanic respondents had significantly higher odds of experiencing one or more ongoing symptoms compared to non-Hispanic White respondents (AOR: 1.79, 95% CI: 1.27–2.53, p < 0.05) and two or more ongoing

						symptoms (AOR: 2.03, 95% CI: 1.45–2.86, p < 0.05).
15	Ferreira et al., 2022	Brazil	Cohort	Hospitalized COVID-19 survivors (18+ yrs)	749 individuals	Female sex was linked to higher dyspnoea (OR: -0.39, 95% CI: -0.55 to -0.23, p < .001), lower functional status (OR: -0.39, 95% CI: -0.56 to -0.23, p < .001), increased fatigue (OR: 4.79, 95% CI: 3.37–6.20, p < .001), and higher anxiety and depression scores (OR: -4.93, 95% CI: -6.24 to -3.61, p < .001). Younger age was associated with higher anxiety and depression (OR: -0.08, 95% CI: -0.14 to 0.01, p = .02). Lower socioeconomic position increased dyspnoea (OR: 0.31, 95% CI: 0.13–0.50, p < .001), fatigue (OR: -2.66, 95% CI: -5.27 to -0.06, p = .05), and worsened functional status (OR: 0.38, 95% CI: 0.08–0.69, p = .01)
16	Feter et al., 2023	Brazil	Cross-sectional	Adults (18+ yrs) COVID-19 positive via PCR/rapid test	2,545 individuals	Women more likely to experience long COVID than men (PR= 1.19 95% CI: 1.08–1.31, p < 0.001) and reported higher rates of fatigue (PR = 1.63, 95% CI: 1.33–2.01, p < 0.05.), neurological complications (PR = 1.56, 95% CI: 1.25–1.94, p < 0.05), headaches (PR = 1.52, 95% CI: 1.22–1.90, p < 0.05), and hair loss (PR = 4.47, 95% CI: 2.97–6.73, p < 0.05).

17	Evering et al., 2023	United States	Cohort	Non-hospitalized adults (18+ yrs) with mild-to-moderate COVID-19	506 participants included in the analysis out of 1162 who received bamlanivimab	Non-Hispanic ethnicity (aRR= 1.92, 95% CI 1.19–3.13, p = 0.007) and females (aRR=1.91 95% CI 1.28–2.85; p = 0.001) were associated with long COVID. Hispanic/Latino ethnicity with lower risk (aRR= 0.52 95% CI, 0.32–.84, p=0.007)
18	Fisher et al., 2024	United States	Cross-sectional	Adults (18+ yrs) with/without COVID-19 history	2,828 individuals	Older age was associated with greater awareness and likelihood of seeking care for long COVID (OR = 1.03 per year; 95% CI: 1.01–1.05; p = 0.013). Higher awareness was also observed among those with a master's degree or higher versus no high school diploma (OR = 2.21; 95% CI: 1.49–3.26; p < 0.001), and among respondents earning ≥$75,000 compared to <$10,000 (OR = 2.34; 95% CI: 1.49–3.70; p < 0.001). Black respondents (OR = 0.64; 95% CI: 0.51–0.81; p < 0.001) and Hispanic respondents—especially those completing the survey in Spanish (OR = 0.31; 95% CI: 0.23–0.41)—had lower odds of awareness compared to English-speaking respondents (OR = 0.59; 95% CI: 0.46–0.76; p < 0.001).
19	Forster et al., 2022	Germany	Cohort	Adults (18+ yrs) with confirmed SARS-CoV-2	1459 individuals	Female sex associated with an increased risk of long COVID (OR =1.81 95% CI 1.45, 2.24, p < 0.001).

						Individuals with higher educational qualifications had a higher likelihood of reporting long COVID symptoms (OR= 1.147 95% CI: 1.057; 1.245, p = 0.001)
20	Hejazian et al., 2024	United States	Cross-sectional	Adults (18+ yrs) with confirmed SARS-CoV-2 infection	108,455 individuals	Individuals aged 35-44 years (aOR = 1.1, 95% CI: 1.0-1.2, p = 0.035) and 45-64 years (aOR = 1.1, 95% CI: 1.0-1.2, p = 0.030) , and females (aOR = 1.6, 95% CI: 1.5-1.7, p < 0.001) have increased odds of post-COVID conditions, while those aged >64 years had a lower likelihood (aOR = 0.8, 95% CI: 0.7-0.9, p < 0.001). Black individuals (aOR = 0.8, 95% CI: 0.7-0.9, p = 0.004) and an annual household income ≥$100k (aOR = 0.8, 95% CI: 0.7-0.9, < 0.001) were associated with lower odds experiencing long COVID.
21	Heller et al., 2022	Israel	Cross-sectional	Adults (18+ yrs) from marginalized groups with/without prior COVID-19	2,246 individuals	Arab Israelis reported more symptoms after moderate/severe acute COVID-19, increasing from 1.7 to 3.2 symptoms (p < 0.05). Non-Ultra-Orthodox Jews saw higher rates of fatigue (33.9% to 53.9%, p < 0.10), loss of taste/smell (2.6% to 19.3%, p < 0.05), and muscle/joint pain (14.5% to 30.6%, p < 0.10). Ultra-Orthodox Jews had a rise in long COVID prevalence from 37.5% to 76.1% (p < 0.05). Low-income individuals had a higher

						baseline symptom prevalence (55.5% vs. 42.1% in high-income, p < 0.01). In high-income individuals, fatigue increased from 29.0% to 58.0% (p < 0.05), and loss of taste/smell rose from 1.8% to 26.0% (p < 0.05)
22	Henderson et al., 2024	United Kingdom	Cohort	Adults (18+ yrs) with/without COVID-19 history	19,462,260 individuals	Crude incidence rates of long COVID were higher among women (177.5 95% CI: 175.5–179). Additionally, the highest rates were observed in 40–49-year-olds (205.5, 95% CI: 202–209) and 50–59-year-olds (194, 95% CI: 191–197.5) and white individuals (151, 95% CI: 150–152.5)
23	Hetlevik et al., 2023	Norway	Cohort	Adults (18+ yrs) SARS-CoV-2 positive	539,603 individuals	Female sex was associated with an increased risk, particularly for fatigue (RR 2.04, 95% CI: 1.89–2.20, p = 0.0951) and shortness of breath (RR 1.33, 95% CI: 1.20–1.49, p = 0.0217). Individuals aged 60 and older had a higher risk of shortness of breath (RR 2.04, 95% CI: 1.65–2.52, p = 0.0002)
24	Hossain et al., 2021	Bangladesh	Cohort	Adults (18+ yrs) COVID-19 survivors, mostly non-hospitalized	2198 individuals	Rural residence associated with the presence of long COVID (OR = 1.51; 95% CI: 1.16–1.98; p = 0.002). Females were associated with increased severity of long COVID (β = 0.064; 95% CI: 0.194–0.262; p = 0.010). Frontline workers (healthcare professionals and police) were associated with greater

						severity of long COVID (β = 0.123; 95% CI: 0.206–0.407; p < 0.001).
25	Krishnadath et al., 2023	Suriname	Cohort study	Adults (18+ yrs) COVID-19 positive	106 individuals	Women had a higher risk of experiencing at least one persisting symptom 3–4 months after COVID-19 (RR = 2.3; 95% CI: 1.00–5.4)
26	Linh et al., 2024	Vietnam	Cross-sectional	Children/adolescents (6–16 yrs) diagnosed with COVID-19	422 individuals	Girls were more likely to report long COVID (OR 1.25, 95% CI 1.15–1.37; p <0.001). Children aged 6–9 years were at higher risk (OR 1.24, 95% CI 1.12–1.37; p<0.001).
27	Mahmoodi et al. 2023,	Iran	Cross-sectional	General population (15–78 yrs) with confirmed COVID-19	308 individuals	Participants with education above a bachelor's degree (OR: 3.747, 95% CI: 1.587–8.844, P=0.003) and females (OR: 2.725, 95% CI: 1.422–5.221, P=0.003) had higher odds of experiencing long COVID symptoms.
28	Martinez -Ayala et al., 2023	Colombia	Cohort	Adults (median age 53) with confirmed COVID-19, including those hospitalized	1,723 individuals	Female sex was significantly associated with Long COVID severity (RR 1.148, 95% CI [1.038–1.268], p=0.007).
29	Martin et al., 2024	United States	Cross-sectional	Adults (18+ yrs) recovering from severe COVID-19 in rehab facilities	48 individuals	Higher SF-36 QoL scores were linked to male gender (OR = 26.4, p = 0.002, 95% CI = [3.23, 215.59]), income ≥$70,000 (OR = 8.1, p = 0.004, 95% CI = [1.06, 62.45]), and urban residency (OR = 7.8, p = 0.046, 95% CI = [1.04, 58.55]).

30	Paranhos et al., 2022	Brazil	Cross-sectional	Adults (18+ yrs) with long COVID and neuro symptoms	219 individuals (139 chronic olfactory dysfunction)	Ageusia was strongly associated with olfactory dysfunction (OR = 13.24, 95% CI: 5.24–33.47, p < 0.001) and with severe olfactory dysfunction (OR = 2.82, 95% CI: 1.53–5.21, p < 0.001).
31	Merzon et al., 2022	Israel	Cross-sectional	Children (5–18 yrs) SARS-CoV-2 positive	20,601 children	Older children (specifically those closer to 15 years old) were significantly more likely to develop long COVID (OR = 1.38, 95% CI = 1.24–1.54, p < 0.001) compared to younger children.
32	Miyazato et al., 2022	Japan	Cross-sectional	Japanese adults (median age 47) recovered from COVID-19	457 Individuals	Women were more likely to experience long COVID symptoms such as fatigue (OR: 2.03, 95% CI: 1.31–3.14, p = 0.001), dysosmia (OR: 1.91, 95% CI: 1.24–2.93, p = 0.003), dysgeusia (OR: 1.56, 95% CI: 1.02–2.39, p = 0.042), and hair loss (OR: 3.00, 95% CI: 1.77–5.09, p < 0.001) and persistence of any symptom (coefficient: 38.0, 95% CI: 13.3–62.8, p = 0.003).Younger age was inversely associated with dysosmia (OR: 0.96 per year of age, 95% CI: 0.94–0.98, p < 0.001) and dysgeusia (OR: 0.98 per year of age, 95% CI: 0.96–1.00, p = 0.015)
33	Mkoma et al., 2024	Denmark	Cohort	Adults (18+ yrs) diagnosed with COVID-19	2,287,175 individuals	Ethnic minorities from North Africa (aHR = 1.41, 95% CI = 1.12–1.79, p = 0.003), the Middle East (aHR = 1.38, 95% CI = 1.24–1.55, p < 0.001), Eastern Europe (aHR = 1.35, 95% CI = 1.22–

						1.49, p < 0.01), and Asia (aHR = 1.23, 95% CI = 1.09–1.40, p = 0.001) had a significantly greater risk of being diagnosed with long COVID compared to native Danes.
34	Modji et al., 2024	United States	Cohort	Workers (16+ yrs) compensated for COVID-19-related work loss	2001 individuals	Higher odds of long COVID were associated with older age (OR = 2.35, 95% CI = 1.72–3.24, p < 0.001) and non-White race (OR = 1.75, 95% CI = 1.22–2.50, p = 0.002). Workers in metropolitan areas (aOR = 1.97, 95% CI = 1.15–3.59, p = 0.019), public administration (aOR = 2.13, 95% CI = 1.11–3.94, p = 0.018), and manufacturing (aOR = 2.44, 95% CI = 1.08–5.19, p = 0.025) also had increased risk
35	Mukherjee et al., 2022	United States	Cohort	Individuals (all ages) with COVID-19	1,371,110 individuals	Older age and females were significantly associated with higher odds of developing long COVID symptoms statistically significant after Bonferroni correction (p < 0.0014)
36	Laughlin et al., 2023	United States	Cohort	Adults (18+ yrs) including SARS-CoV-2-positive and negative individuals	3,401 individuals	Hispanic participants had higher odds of reporting fair/poor health than non-Hispanics (OR = 1.94; 95% CI: 1.36–2.78; p < 0.01). At 3 months, participants of Other/Multiple races (OR = 1.90; 95% CI: 1.25–2.88; p < 0.01) and by 6 months, Asian (OR = 1.88; 95% CI: 1.13–3.12; p = 0.02) and Other/Multiple race participants (OR =

						1.83; 95% CI: 1.10–3.05; p = 0.02) were more likely to report fair/poor health than White participants.<br><br>Hispanic participants reported more headaches at 3 months (OR = 1.70; 95% CI: 1.20–2.42; p < 0.01), and at 6 months, higher odds of headaches (OR = 1.97; 95% CI: 1.25–3.11; p < 0.01), nausea/vomiting (OR = 2.20; 95% CI: 1.02–4.77; p = 0.05), and other symptoms (OR = 2.32; 95% CI: 1.14–4.72; p = 0.02).<br><br>At 3 months, Asian participants had more sore throats (OR = 1.72; 95% CI: 1.09–2.72; p = 0.02) and less shortness of breath (OR = 0.32; 95% CI: 0.14–0.74; p < 0.01). Black participants were less likely to report loss of smell (OR = 0.45; 95% CI: 0.25–0.82; p < 0.01), wheezing (OR = 0.29; 95% CI: 0.09–0.98; p = 0.05), and other symptoms (OR = 0.35; 95% CI: 0.13–0.95; p = 0.04).<br><br>Participants of Other/Multiple races had higher odds of wheezing at 3 months (OR = 2.22; 95% CI: 1.04–4.75; p = 0.04) and lower odds of loss of

						smell at 6 months (OR = 0.23; 95% CI: 0.05–0.95; p = 0.04).
37	Pastorello et al., 2025	France	Cohort	Individuals (15+ yrs) with probable symptomatic COVID-19	82,616 individuals	The risk of long COVID was higher among women (aRR = 1.33, 95% CI: 1.16–1.54, p < 0.001) and those with a vocational diploma (aRR = 1.37, 95% CI: 1.12–1.69, p = 0.026).
38	Pelà et al., 2022	Italy	Cohort	Adults (18+ yrs) with confirmed COVID-19 (inpatient or outpatient)	223 individuals	Women had higher odds of experiencing persistent symptoms dyspnea (OR = 2.357, 95% CI: 1.124–4.944, p = 0.023), fatigue (OR = 6.726, 95% CI: 2.349–19.263, p = 0.000), chest pain (OR = 2.046, 95% CI: 1.006–4.158, p = 0.048), and palpitations (OR = 2.307, 95% CI: 1.142–4.658, p = 0.020). Younger patients reported higher prevalence of fatigue (OR = 0.958, 95% CI: 0.923–0.995, p = 0.025), chest pain (OR = 0.963, 95% CI: 0.934–0.994, p = 0.019), and palpitations (OR = 0.969, 95% CI: 0.938–1.000, p = 0.051) at follow-up.
39	Perlis et al., 2022	United States	Cross-sectional	Adults (18+ yrs) with self-reported positive COVID-19 test (2 months prior)	16,091 individuals	Older individuals had higher risk per decade after 40 years (OR = 1.15, 95% CI: 1.12-1.19, p < 0.001). Women were

						more likely to develop long COVID (OR = 1.91, 95% CI: 1.73-2.13, p < 0.001) and had higher burden of symptoms (OR = 0.74, 95% CI: 0.64-0.86, p < 0.001) . Higher education reduced the risk (graduate vs high school OR = 0.67, 95% CI: 0.56-0.79, p < 0.001). Urban residence was protective (OR = 0.74, 95% CI: 0.64-0.86, p < 0.001).
40	Loannou et al., 2022	United States	Cohort study	Adults (18+ yrs) with confirmed SARS-CoV-2 infection, 3+ months post-infection	198,601 individuals	Long COVID was more frequently documented among Black (AOR = 1.10, 95% CI: 1.08–1.21), American Indian/Alaska Native (AOR = 1.18, 95% CI: 1.03–1.35), and Hispanic individuals (AOR = 1.15, 95% CI: 1.10–1.21). Documentation rates increased with age, peaking at 80–84 years (AOR = 1.38, 95% CI: 1.28–1.48) before declining slightly in those ≥90 years (AOR = 1.21, 95% CI: 1.09–1.34). Urban residents were also more likely to receive long COVID care than rural ones (AOR = 1.14, 95% CI: 1.10–1.19).
41	Jacobs et al., 2023	United States	Cross-sectional	Adults (18+ yrs) including SARS-CoV-2 positive individuals	257,554 individuals	Blacks (OR = 1.10, 95% CI: 1.03–1.16, p < 0.0001), Hispanics (OR = 1.35, 95% CI: 1.29–1.41, p < 0.0001), and females (OR = 1.85, 95% CI: 1.79–1.91, p < 0.0001) had significantly higher odds of developing long COVID. Cognitive symptoms were more likely among individuals living in the South (OR =

						1.25, 95% CI: 1.14–1.36, p < 0.0001). Memory difficulties were more common among Blacks (OR = 1.88, 95% CI: 1.77–1.81, p < 0.0001) and females (OR = 1.34, 95% CI: 1.24–1.45, p < 0.0001), while difficulty understanding was more likely among Hispanics (OR = 1.83, 95% CI: 1.41–2.36, p < 0.0001)
42	Qasmieh et al., 2023	United States	Cross-sectional	Adults (18+ yrs) with current or previous SARS-CoV-2 infection	3042 individuals	Females were more likely to report long COVID symptoms than males (aPR: 1.8, 95% CI: 1.4–2.3)
43	Quaranta et al., 2023	Italy	Cohort	Adults (18+ yrs) with COVID-19 (hospitalized or outpatient)	436 individuals	Female sex was a significant predictor (OR= 0.513 95% CI 0.316–0.833, p = 0.007)
44	Ramírez-Toscano et al., 2024	Mexico	Cross-sectional	Adults (20+ yrs) with confirmed COVID-19	869 individuals	Long COVID was more prevalent in women (41.7%, 95% CI: 36.8–46.8). Prevalence increased with age, from 33.4% (95% CI: 27.6–39.9) in the 20–39 age group to 42.5% (95% CI: 31.2–54.6) in those aged 60 and older. Individuals living in rural areas had a higher prevalence (48.8%, 95% CI: 37.9–59.8), as did those with middle school education (42.0%, 95% CI: 34.7–49.6). Long COVID prevalence decreased with increasing socioeconomic level, from 49.9% (95% CI: 40.0–59.8) in low-income individuals to 33.0% (95% CI: 26.2–40.5) in those with higher income.

45	Resendez et al., 2024	United States	Cohort	Adults (18+ yrs) with prior SARS-CoV-2 infection	367,148 individuals	Patients with long COVID had a mean age of 60.85 years (95% CI: 60.79–60.91), compared to 52.14 years (95% CI: 52.03–52.24) for those without long COVID. Men were disproportionately affected, making up 87.48% of long COVID cases vs. 76.31% in non-long COVID cases (P<.001), women accounted for only 11.8% of long COVID cases compared to 17.05% in the non-long COVID group (P<0.001)
46	Robertson et al., 2023	United States	Cross-sectional	Adults (18+ yrs) with and without prior SARS-CoV-2 infection	3,042 individuals	Higher prevalence was observed in women (aPR: 1.84, 95% CI: 1.40–2.42), individuals with annual income <$60,000 (aPR: 2.22, 95% CI: 1.31–3.78), and those who were employed (aPR: 1.34, 95% CI: 1.02–1.76).However, prevalence was lower among Non-Hispanic Black (aPR: 0.60, 95% CI: 0.38–0.96), Hispanic (aPR: 0.57, 95% CI: 0.37–0.89), Asian/Pacific Islander (aPR: 0.44, 95% CI: 0.21–0.93) individuals, and adults aged ≥65 (aPR: 0.43, 95% CI: 0.27–0.66)
47	Rocha et al., 2024	Brazil	Cohort	Adults (18+ yrs) hospitalized for COVID-19 and discharged	259 individuals	The prevalence was higher among individuals of older age, lower income, hypertension (p < 0.01), and diabetes (p < 0.01). Patients with longer hospital stays had a higher prevalence of post-COVID-19 syndrome (p < 0.01). At 12 months, the presence of three or

						more symptoms was significantly higher among female patients (p < 0.01). Fatigue was the most common symptom at six months (55.3%) and 12 months (40.6%), followed by memory problems (36.8%; 20%) and hair loss (26.8%; 11.2%). Those with no job at 6 months (unemployed) had 94.6% of reporting long COVID symptoms (p<0.01) and those with a job 79.8% reported symptoms
48	Romero-Rodríguez et al., 2023	Spain	Cross-sectional	Healthcare professionals (14+ yrs) with varying COVID-19 status including long COVID	1,490 individuals	Women were more likely to develop long COVID (OR: 1.90, 95% CI: 1.32–2.74, p = 0.001)
49	Wu et al., 2024	United States	Cross-sectional	Adults (18+ yrs) with reported positive COVID-19 test	134,444 individuals (124,313 Behavioral Risk Factor Surveillance System, 10,131 from National Health Interview Survey)	Females had higher odds of long COVID (BRFSS: OR = 1.69, 95% CI = 1.64–1.73, p < 0.001; NHIS: OR = 1.48, 95% CI = 1.32–1.66, p < 0.001). Risk was greatest among adults aged 45–54 (BRFSS: OR = 1.56, 95% CI = 1.47–1.66, p < 0.001; NHIS: OR = 1.47, 95% CI = 1.16–1.85, p < 0.001), and those with lower education (BRFSS: OR = 1.41, 95% CI = 1.37–1.45, p < 0.001; NHIS: OR = 1.23, 95% CI = 1.09–1.38, p < 0.001). Lower odds were observed among non-Hispanic Black (BRFSS: OR = 0.82, 95% CI = 0.77–0.86, p < 0.001; NHIS: OR = 0.83, 95% CI = 0.68–1.00, p < 0.001) and Asian individuals (BRFSS:

						OR = 0.56, 95% CI = 0.50–0.62, p < 0.001; NHIS: OR = 0.49, 95% CI = 0.35–0.68, p < 0.001).
50	Shabnam et al., 2023	United Kingdom	Cohort	Adults (16+ yrs) with confirmed SARS-CoV-2 infection	201,799 individuals	Individuals in the most deprived decile (aOR = 1.46, 95% CI = 1.34–1.59), women (aOR = 1.56, 95% CI = 1.40–1.73) patient-facing healthcare roles (aOR = 1.76, 95% CI = 1.27–2.44) and education (aOR = 1.68, 95% CI = 1.31–2.16) had a higher risk of long COVID
51	Shigematsu et al., 2024	Japan	Cohort	Hospitalized adults (18+ yrs) with confirmed COVID-19	1,009 individuals	Older age was associated with memory impairment (OR = 1.024, 95% CI: 1.010–1.040, p = 0.009)
52	Silva et al.,2023	Brazil	Cohort	General population (avg. age ~40) with confirmed SARS-CoV-2 infection	1,371 individuals	Female (IRR: 1.60, 95% CI: 1.44–1.77, p < 0.001) and non-White race (IRR: 1.14, 95% CI: 1.00–1.30, p = 0.04) were predictors of a higher number of symptoms in post-COVID-19 syndrome and were strongly associated with long-term symptoms
53	Slurink et al., 2024	Netherlands	Cohort	Adults (18+ yrs) with long COVID, prior COVID-19 without symptoms, or never infected	3022 individuals	Older age (OR= 1.13; 95% CI 1.03-1.26; p < 0.05) and Western ethnicity (OR= 1.88; 95% CI 1.49-2.41; p < 0.001) were predictors of long COVID
54	Song and Giuriato 2023	United States	Cohort	Adults (18+ yrs) classified as narrow/broad long haulers or non–long haulers	816,027 individuals	Long haulers were more likely to be older (mean age: 56.6 years for narrow-definition, 53.7 years for broad-definition, vs. 46.4 years for non–long haulers, p < 0.001) and female (58.1% narrow-definition,

						59.7% broad-definition, vs. 50.9% non–long haulers, p < 0.001). Non-Hispanic White individuals making up a larger proportion of long haulers (64.1% narrow-definition, 61.3% broad-definition, vs. 60.9% non–long haulers, p < 0.001), while Black, Hispanic/Latino, and Asian individuals were slightly underrepresented. Long haulers were less likely to reside in metropolitan areas (82.7% narrow-definition, 85.1% broad-definition, vs. 86.7% non–long haulers, p < 0.001)
55	Stephens et al., 2024	United States	Cohort	Veteran affair patients (18+ yrs) with prior SARS-CoV-2 infection, including those with and without long COVID	363,825 individuals	Non-Hispanic Black Veterans had higher odds of long COVID compared to non-Hispanic Whites (OR = 1.14, 95% CI = 1.12–1.16, p < 0.001), as did Hispanic individuals (OR = 1.09, 95% CI = 1.06–1.12, p < 0.001), while Asian individuals had slightly lower odds (OR = 0.95, 95% CI = 0.89–1.02, p = NS). Veterans with only vocational or technical school education had increased risk (aOR = 1.10, 95% CI = 1.04–1.18, p < 0.001) compared to high school graduates, whereas those with graduate degrees had lower odds (aOR = 0.94, 95% CI = 0.92–0.96, p < 0.001). Urban residents were slightly more likely to experience long COVID

						than rural residents (aOR = 1.04, 95% CI = 1.02–1.06, p < 0.001)
56	Subramanian et al., 2022	United Kingdom	Cohort	Non-hospitalized adults (18+ yrs) with SARS-CoV-2	2,430,729 individuals	Individuals at higher risk of long COVID included women (aHR: 1.52, 95% CI: 1.48–1.56), those of Black Afro-Caribbean (aHR: 1.21, 95% CI: 1.10–1.34) and Mixed ethnicity (aHR: 1.14, 95% CI: 1.07–1.22), and people living in the most deprived areas (aHR: 1.11, 95% CI: 1.07–1.16). In contrast, older adults (≥70 years) had a lower risk (aHR: 0.75, 95% CI: 0.70–0.81).
57	Terai et al., 2023	Japan	Cohort	Hospitalized adult COVID-19 patients (18+ yrs)	1,200 individuals	Higher odds of long COVID were observed in women (OR: 2.54, 95% CI: 1.43–4.53, p = 0.0015) and middle-aged adults (41–64 years) (OR: 3.60, 95% CI: 1.92–6.78, p < 0.0001).
58	Van Cleve et al., 2024	United States	Cross-sectional	US veterans (18+ yrs) with confirmed COVID-19	658 veterans	Greater risk of long COVID was associated with increasing age (OR: 1.02, 95% CI: 1.003–1.042, p = 0.023) and being female (OR: 1.84, 95% CI: 1.018–3.330, p = 0.043). In contrast, higher education was protective: veterans with a master's degree had lower odds of long COVID (OR: 0.40, 95% CI: 0.198–0.818, p = 0.012), those with some college or an associate degree were less likely to experience

						moderate symptoms (OR: 0.41, 95% CI: 0.20–0.86, p = 0.018), and those with a bachelor's degree were less likely to experience severe symptoms (OR: 0.44, 95% CI: 0.20–0.99, p = 0.047).
59	Wander et al., 2023	United States	Cohort	US veterans aged(18+yrs) with confirmed SARS-CoV-2	388,980 individuals	Higher likelihood of U09.9 documentation was observed among individuals aged ≥90 years (AHR: 1.67, 95% CI: 1.52–1.84), women (AHR: 1.23, 95% CI: 1.18–1.29), and Hispanic individuals (AHR: 1.33, 95% CI: 1.27–1.39). Black individuals (AHR: 0.72, 95% CI: 0.69–0.75) and urban residents (AHR: 0.90, 95% CI: 0.86–0.94) were less likely to have documentation.
60	Wang et al., 2024	United Kingdom	Cohort	Adults (18+ yrs) with confirmed SARS-CoV-2 infection	1,554,040 individuals	Higher risk of long COVID was observed among females (AHR: 1.171, 95% CI: 1.156–1.186, p < 0.001) and non-White individuals (AHR: 1.145, 95% CI: 1.130–1.161, p < 0.001), while those living in the most deprived areas had a lower likelihood (AHR: 0.955, 95% CI: 0.937–0.974, p < 0.001).
61	Wang et al., 2024	China	Cross - sectional	Older adults (60+ yrs) with confirmed SARS-CoV-2 infection	4804 individuals	Low economic status was associated with increased risk (adjusted OR: 1.48, 95% CI: 1.22–1.82, p < 0.001), with the effect being significant in males (adjusted OR: 1.98, 95% CI: 1.42–2.76) but not in females (adjusted OR: 1.21,

						95% CI: 0.95–1.56). Additionally, ethnic minority participants had a higher risk of developing long COVID symptoms (adjusted OR: 3.38, 95% CI: 1.87–6.29).
62	Wilk et al., 2023	27 European countries	Cohort	Adults (50+ yrs) who reported either a positive COVID-19 test or symptoms attributed to COVID-19 without a test	4,004 individuals	Lower risk of long COVID was associated with higher education (OR: 0.72, 95% CI: 0.52–0.99, p = 0.05) and being male (OR: 0.69, 95% CI: 0.52–0.92, p = 0.01), while employment was linked to a higher risk (OR: 1.53, 95% CI: 1.18–1.98, p = 0.00). Additionally, older age was associated with fewer symptoms (RR: 0.92, 95% CI: 0.86–0.98, p = 0.01).
63	Wong et al., 2023	China	Cross - sectional	Adults (18+ yrs) with confirmed history of COVID-19	2,712 individuals	Severe long COVID was associated with female gender (aOR: 1.49, 95% CI: 1.13–1.95, p = 0.004), and employment in transportation/logistics/disciplinary sectors (aOR: 2.52, 95% CI: 1.58–4.03, p < 0.001)
64	Pfaff et al., 2023	United States	Cohort	All ages diagnosed with long COVID (ICD-10-CM code U09.9)	33,782 individuals	Women were more likely to receive a U09.9 diagnosis than men (66.9% vs. 55.6%). White individuals were overrepresented among long COVID diagnoses compared to all COVID-positive patients (71.8% vs. 61.1%, p < 0.01), as were non-Hispanic individuals (79.6% vs. 74.8%, p < 0.01).
65	Bai et al., 2022	Italy	Cohort	Hospitalized adults (median age 57) with	377 individuals	Higher risk of long COVID was associated with female sex (AOR: 3.32,

				confirmed SARS-CoV-2 infection and documented virological clearance		95% CI: 1.78–6.17, p < 0.0001) and older age, with risk increasing per 10-year increment (AOR: 1.03, 95% CI: 1.01–1.05, p = 0.01).
66	Dryden et al., 2022	South Africa	Cohort	Adults (18+ yrs) hospitalized with confirmed SARS-CoV-2 infection and later discharged	1873 individuals	Female sex was associated with new or persistent symptoms (aIRR: 1.20, 95% CI: 1.04–1.38, p < 0.05) and breathlessness (aIRR: 1.37, 95% CI: 1.05–1.77). Compared to Black individuals, White (aIRR: 1.86, 95% CI: 1.32–2.63) and Mixed ethnicity individuals (aIRR: 1.69, 95% CI: 1.04–2.76) were more likely to report breathlessness, while White (aIRR: 1.84, 95% CI: 1.24–2.70), Mixed (aIRR: 1.95, 95% CI: 1.10–3.45), and Indian individuals (aIRR: 1.89, 95% CI: 1.02–3.52) were more likely to report non-recovery.
67	Asadi-Pooya et al., 2021	Iran	Cohort	Hospitalized adults (18+ yrs) with confirmed COVID-19	4681 individuals	Women were more likely to experience long COVID (OR: 1.268, 95% CI: 1.122–1.432, p = 0.0001)
68	Pazukhina et al., 2022	Russia	Cohort	Hospitalized patients with confirmed COVID-19, iadults (median age 56.8 years, IQR 47.0–65.8) and children (median age 9.5 years, IQR 2.4–14.8).	1373 individuals	Female sex was associated with long COVID at 6-month and 12-month follow-up (OR 2.04, 95% CI 1.57–2.65) and (OR 2.04, 95% CI 1.54–2.69)
69	Moy et al., 2022	Malaysia	Cross-sectional	Adults (18+ yrs) with confirmed history of COVID-19	732 individuals	Females had a higher odd of experiencing long COVID (OR: 1.58 95%CI 1.02- 2.45)

70	Khullar et al., 2023	United States	Cohort	Adults (20+ yrs) with and without COVID-19, across inpatient and outpatient settings	310,220 individuals	hospitalized Black patients had higher odds of being diagnosed with diabetes (adjusted odds ratio [OR]: 1.96, 95% confidence interval [CI]: 1.50—2.56, q<0.001) and headaches (OR: 1.52, 95% CI: 1.11—2.08, q=0.02), compared to hospitalized white patients. Hospitalized Hispanic patients had higher odds of headaches (OR: 1.62, 95% CI: 1.21—2.17, q=0.003) and dyspnea (OR: 1.22, 95% CI: 1.05—1.42, q=0.02), compared to hospitalized white patients. Among nonhospitalized patients, Black patients had higher odds of being diagnosed with pulmonary embolism (OR: 1.68, 95% CI: 1.20—2.36, q=0.009) and diabetes (OR: 2.13, 95% CI: 1.75—2.58, q<0.001), but lower odds of encephalopathy (OR: 0.58, 95% CI: 0.45—0.75, q<0.001), compared to white patients. Nonhospitalized Hispanic patients had higher odds of being diagnosed with headaches (OR: 1.41, 95% CI: 1.24—1.60, q<0.001) and chest pain (OR: 1.50, 95% CI: 1.35—1.67, q < 0.001), but lower odds of encephalopathy (OR: 0.64, 95% CI: 0.51—0.80, q<0.001). greater severity of long COVID burden among racial/ethnic minority groups,

						particularly Black and Hispanic populations
71	Sharif-Askari et al., 2024	United Arab Emirates	Cohort	Non-hospitalized adults (18+ yrs) with mild-to-moderate COVID-19 symptoms	28,375 individuals	Females (aHR 1.28 95% CI 1.002-1.56, p=0.048) and Caucasians (aHR:2.88 95% CI 2.09-3.99, p<0.001) are at higher risk of developing long COVID during the pre-Omicron period
